# Quasi-Solid-State Ion-Conducting Arrays Composite Electrolytes with Fast Ion Transport Vertical-Aligned Interfaces for All-Weather Practical Lithium-Metal Batteries

**DOI:** 10.1007/s40820-022-00952-z

**Published:** 2022-10-31

**Authors:** Xinyang Li, Yong Wang, Kai Xi, Wei Yu, Jie Feng, Guoxin Gao, Hu Wu, Qiu Jiang, Amr Abdelkader, Weibo Hua, Guiming Zhong, Shujiang Ding

**Affiliations:** 1 School of Chemistry, Xi’an Key Laboratory of Sustainable Energy Materials Chemistry, State Key Laboratory of Electrical Insulation and Power Equipment, Xi’an Jiaotong University, Xi’an, 710049 People’s Republic of China; 2 State Key Laboratory for Mechanical Behaviour of Materials, Xi’an Jiaotong University, Xi’an, 710049 People’s Republic of China; 3 Yangtze Delta Region Institute (Huzhou), University of Electronic Science and Technology of China, Huzhou, Zhejiang 313001 People’s Republic of China; 4 School of Materials and Energy, University of Electronic Science and Technology of China, Chengdu, 610054 People’s Republic of China; 5 Faculty of Science and Technology, Bournemouth University, Talbot Campus, Fern Barrow, Poole, BH12 5BB UK; 6 Institute for Applied Materials-Energy Storage Systems (IAM-ESS), Karlsruhe Institute of Technology (KIT), 76344 Eggenstein-Leopoldshafen, Germany; 7 School of Chemical Engineering and Technology, Xi’an Jiaotong University, Xi’an, Shaanxi, 710049 People’s Republic of China; 8 Laboratory of Advanced Spectroelectrochemsitry and Li-ion Batteries, Dalian Institute of Chemical Physics, Chinese Academy of Sciences, Dalian, 116023 People’s Republic of China; 9 State Key Laboratory of Organic-inorganic Composites, Beijing University of Chemical Technology, Beijing, 100029 People’s Republic of China

**Keywords:** Solid-state batteries, Composite electrolytes, Vertical-aligned ion-conducting arrays, Interfacial ion-conduction mechanism, All-weather practical electrolyte design

## Abstract

The rapid improvement in the gel polymer electrolytes (GPEs) with high ionic conductivity brought it closer to practical applications in solid-state Li-metal batteries. The combination of solvent and polymer enables quasi-liquid fast ion transport in the GPEs. However, different ion transport capacity between solvent and polymer will cause local nonuniform Li^+^ distribution, leading to severe dendrite growth. In addition, the poor thermal stability of the solvent also limits the operating-temperature window of the electrolytes. Optimizing the ion transport environment and enhancing the thermal stability are two major challenges that hinder the application of GPEs. Here, a strategy by introducing ion-conducting arrays (ICA) is created by vertical-aligned montmorillonite into GPE. Rapid ion transport on the ICA was demonstrated by ^6^Li solid-state nuclear magnetic resonance and synchrotron X-ray diffraction, combined with computer simulations to visualize the transport process. Compared with conventional randomly dispersed fillers, ICA provides continuous interfaces to regulate the ion transport environment and enhances the tolerance of GPEs to extreme temperatures. Therefore, GPE/ICA exhibits high room-temperature ionic conductivity (1.08 mS cm^−1^) and long-term stable Li deposition/stripping cycles (> 1000 h). As a final proof, Li||GPE/ICA||LiFePO_4_ cells exhibit excellent cycle performance at wide temperature range (from 0 to 60 °C), which shows a promising path toward all-weather practical solid-state batteries.

**Supplementary Information:**

The online version contains supplementary material available at 10.1007/s40820-022-00952-z.

## Highlights

- The composite gel electrolyte with low tortuosity ion-conducting arrays (GPE/ICAs) exhibiting high room-temperature ionic conductivity (1.08 mS cm^−1^) was successfully prepared by directionally growing ice crystals and in-situ polymerization.
- The stable and rapid Li^+^ migration through ICAs in the GPE is proved by ^6^Li solid-state nuclear magnetic resonance and synchrotron radiation X-ray diffraction combined with computer simulations.
- Li/LiFePO_4_ full cells using GPE/ICAs exhibit excellent cycle performance and high-capacity retention at wide temperature (0–60 °C), which has the potential towards all-weather practical solid-state batteries.

**Supplementary Information:**

The online version contains supplementary material available at 10.1007/s40820-022-00952-z.

## Graphical abstract

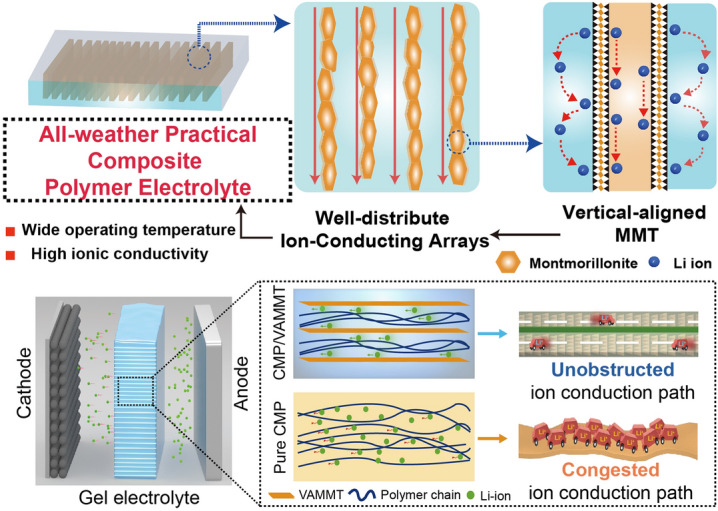

## Introduction

The rapid growth of electric vehicles and portable electronics puts more pressure on the batteries industry to develop high-energy-density and safe devices [1, 2]. Rechargeable solid-state batteries are at the forefront of the proposed storage devices for tomorrow's applications [3, 4]. Solid-state electrolytes have several advantages compared with the liquid electrolytes traditionally used in commercial rechargeable batteries, such as wide electrochemical windows, no risk of flammability or leaking and good thermal stability [5–7]. In addition, many reports show that solid electrolytes could prevent lithium or other metal dendrite growth, thereby realizing the 'holy grail' of high-energy-density metal batteries [8]. Despite the apparent advantages of solid electrolytes, the limited room-temperature ionic conductivity leads to lower capacity utilization and poor rate performance [9]. Solid electrolytes face other challenges, including the sluggish ionic transport at the electrodes/electrolyte interface [10] and the mechanical and chemical instability during cycling [11].

Gel polymer electrolytes (GPEs) as quasi-solid-state electrolytes are considered promising alternatives for liquid electrolytes [12, 13]. GPEs have high room-temperature ionic conductivity [14], excellent interfacial wettability [15] and good mechanical flexibility [16], unlike the rigid ceramic solid electrolytes. However, inhomogeneous ion transport inside the GPEs leads to lithium dendrites growth and deterioration of the battery performance [17]. The irregular ion transport within GPEs results from the rapid ion transport in the free-solvent regions and the slow ion transport in the solvent-polymer synergistic regions [18]. In addition, GPEs generally have poor thermal stability because the solvents in the gel usually have low boiling and high melting points (for example, dimethyl carbonate (DMC), T_b_ = 90 °C, T_m_ = 0.5 °C). The solvents' low thermal stability leaves GPEs with narrow operating temperature windows [19], low ion mobility in the free-solvent region at low temperatures and high gel deformation at high temperatures. The above problems are the main reasons that limit the practical application of GPEs in solid-state Li-metal batteries (SSLMBs).

Several strategies have been adopted to address the problems mentioned above. For example, constructing cross-linked interpenetrating polymer networks by the ultraviolet-curing or thermos-curing methods showed promising results in enhancing the mechanical toughness of GPEs while maintaining their ionic conductivity [20–22]. Also, adding inorganic fillers (SiO_2_ [23], Al_2_O_3_ [24], Li_10_GeP_2_S_12_ [25], Li_7_La_3_Zr_2_O_12_ [26], Li_1.5_Al_0.5_Ge_1.5_(PO_4_)_3_ [27] and others [28, 29]) into GPEs induces additional interface ionic transport and enhances the mechanical strength.

High ionic conductivity can be achieved at the organic–inorganic interface within GPEs due to multiple effects on Li^+^ [30]. Our previous work [31] and also work by Jeon et al. [32] show that layered montmorillonite (MMT) with a high aspect ratio, sizeable interfacial area (Fig. S1) and excellent electrochemical/thermal stability significantly improves the ionic conductivity and mechanical strength of GPEs. However, the random distribution of MMT causes discontinuity in the ionic pathways and also limits the mechanical/thermal stability. Recent studies have shown that vertical-aligned inorganic array structures could improve mechanical strength and create fast ion transport channels inside the polymer matrix [33–35]. For example, Cui et al. showed the enhanced ionic conductivity of polyethene oxide (PEO) electrolytes supported by vertical alumina arrays and polyimide (PI) films with vertical pores [36]. Hu et al. introduced a continuous interface ion transport path by impeding garnet-wood membrane with vertical pores within the polymer matrix [37]. Therefore, we believe that constructing ion-conducting arrays (ICA) with vertically aligned interfaces in GPEs could effectively solve the inhomogeneous ionic transport and also improve the thermal and mechanical stability of GPEs.

**Fig. 1:**
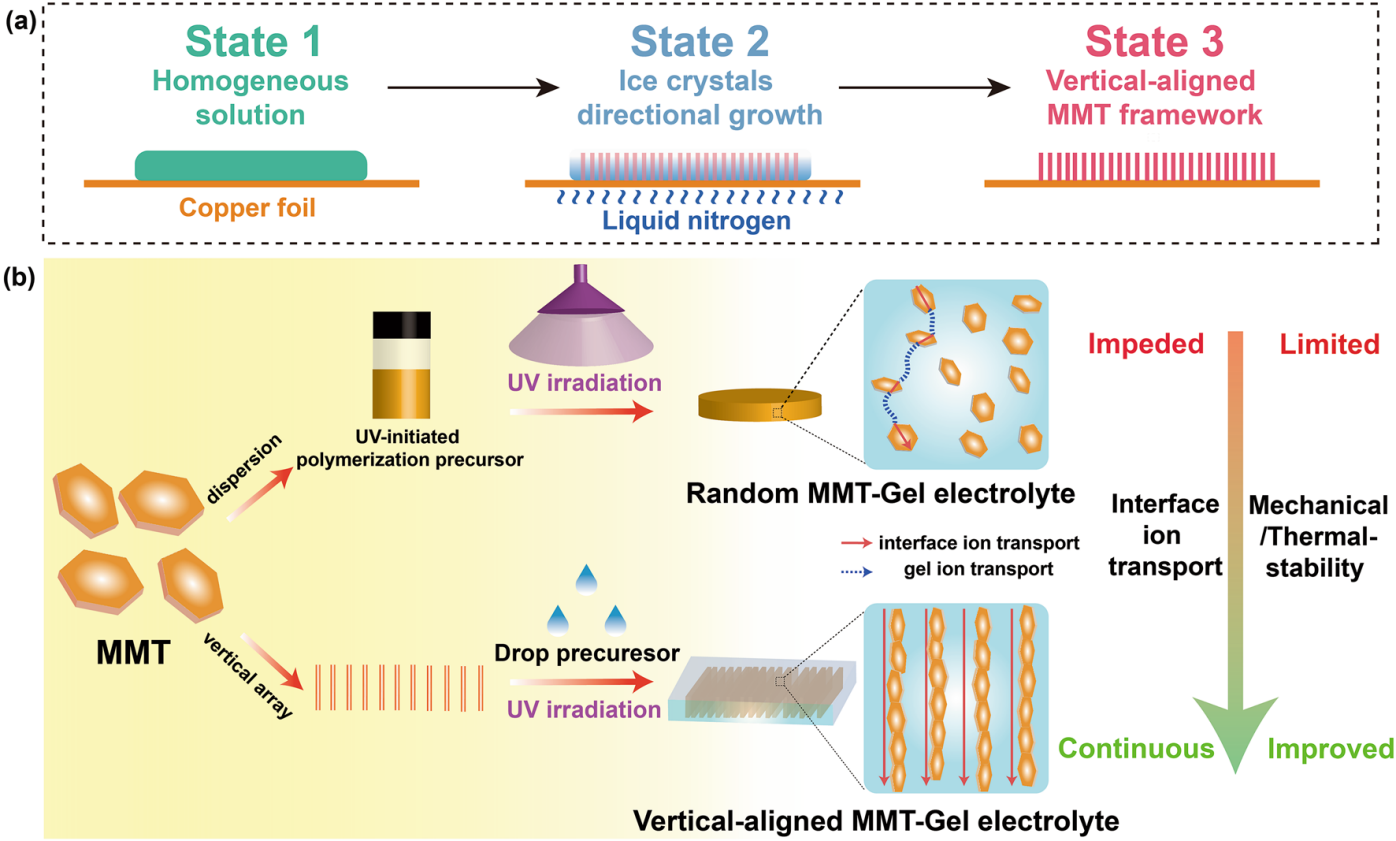
Synthesis of vertical-aligned MMT and composite GPEs with random MMT or VAMMT. **a** Schematic illustrating of VAMMT prepared by directional freezing technology. **b** Schematic of the fabrication process of different GPEs

In this work, we fabricated vertical-aligned MMT arrays as ICA with ultra-low tortuosity by the directional freezing method (Fig. 1a) and created well-distribute continuous ion transport interface in GPEs by UV-induced polymerization to facilitate Li^+^ migration (Fig. 1b). As a result, the GPE/VAMMT exhibits more improved ion transference numbers and higher ionic conductivity, reaching 1.08 mS cm^−1^ at room temperature. The ion transport mechanism in the GPE/VAMMT was investigated using ^6^Li solid-state nuclear magnetic resonance (^6^Li SSNMR), synchrotron X-ray diffraction (SRD) pattern and dynamic computer simulation. The experimental and theoretical results exhibit short ion transport paths with high conductivity at the interface and interlayers of VAMMT, elucidating the fast and homogeneous ion transport. As a result, the Li||LiFePO_4_ full cell with GPE/VAMMT demonstrates a high capacity of ~ 130 mAh g^−1^ and excellent capacity retention of > 85% after 1000 cycling at 30 °C. Even under extreme test temperatures, the Li|| GPE/VAMMT||LiFePO_4_ cells also show a high capacity of ~ 125 mAh g^−1^ with capacity retention of > 95% after 200 cycling at 0 °C and long-term cycling (> 500 cycles) at high temperature (60 °C) with 99.4% Coulombic efficiency. Moreover, the GPE/VAMMT was also tested in Li–S and Li-NCM systems and demonstrated excellent electrochemical performance.

## Experimental and Calculation

### Preparation of Cross-linked Methoxy Poly(ethylene glycol) acrylate (CMP) Liquid Precursor

The raw materials, including liquid electrolyte (1.0 M LiPF_6_ in ethylene carbonate (EC)/diethyl carbonate (DEC) = 1:1 (v/v) with 2.0% LiBOB, Duo Duo chem), were stored in the glove box and used without further purification. One gram of methoxy poly(ethylene glycol) acrylate (MPEGA, Mw≈480, Sigma-Aldrich), 5 mg of poly(ethylene glycol) diacrylate (PEGDA, Mw≈700, Sigma-Aldrich) as cross-linking agent and 5 mg of 1-hydroxycyclohexyl phenyl ketone (HCPK, photoinitiator, Aladdin) (0.5 wt% of MPEGA) were added to 600 μL of liquid electrolyte in the glove box, and a clear solution was obtained after magnetic stirring for 1 h. In the case of the sulfur cathode, the liquid electrolyte in the precursor was replaced with specialized liquid electrolyte in the Li–S system (1.0 M LiTFSI in dioxolane (DOL)/1,2-dimethoxyethane (DME) = 1:1 (v/v) with 1.0% LiNO3, Duo Duo chem).

### Synthesis of VAMMT

For the preparation of VAMMT, montmorillonite (Na-MMT, Shanghai yuanye biotechnology Co. Ltd.) and polyethylene oxide (PEO, *M*_w_≈300,000, Aladdin) as the binder in 2:1 mass ratio were dispersed in the deionized water. A homogeneous solution with 4.5 wt% MMT was obtained by vigorous stirring. The precursor solution was cast onto a clean copper plate using a doctor blade method and freeze-dried at -55 °C for 12 h (using liquid nitrogen for freezing).

### Preparation of CMP/VAMMT

To prepare the composite gel polymer electrolyte with VAMMT, the prepared liquid precursor of CMP was impregnated within VAMMT. Then, VAMMT filled with liquid precursor was irradiated by 365 nm UV lamp for 5 min. Moreover, for the composite gel polymer electrolyte with random MMT filters (CMP/MMT), a certain amount of MMT with 13.2 wt% (total weight of liquid monomer precursor) was added into the liquid precursor of CMP and stirred at 30 °C for 5 h. Then, the mixture was dropped into the cathode and irradiated by the 365 nm UV lamp for 5 min. The pure CMP was fabricated by the same way as CMP/MMT without MMT filters. All the GPEs preparation are conducted in the glove box, in which the O_2_ and H_2_O content were maintained below 0.1 ppm.

### Preparation of Cathode and Battery

Sulfur cathodes were prepared by mixing sulfur (sublimed sulfur, innochem)/Ketjen Black (KB) composites, Super-P and polyvinylidene fluoride (PVDF) in N-methyl pyrrolidone (NMP) to form a slurry at a weight ratio of 8:1:1. After two hours of ball milling. The slurry was coated on a carbon-coated aluminum foil using the doctor blade. Then, the electrodes were dried at 60 ℃ for 8 h in vacuum oven. The sulfur/KB composites were made using a typical melt diffusion process (155 ℃ for 12 h at Ar-filled sealed glass bottle) with a sulfur content of approximately 60 wt%. The sulfur loading was about 0.7 mg cm^−2^. To prepare LiFePO_4_ (LFP) and NCM_523_ cathodes, the active powder, Super-P and polyvinylidene fluoride (PVDF) at a weight ratio of 90:5:5 were ball-milled for 2 h in NMP. The slurry was then cast on aluminum foil and then dried at 120 ℃ for 12 h under vacuum. The loading of cathodes was about 2 mg cm^−2^. All batteries electrochemical evaluation experiments were conducted using coin-type 2025 cells with Li foil as the anode. The Li symmetric cells were assembled by sandwiching the gel electrolytes between two Li metals. The cell assembly was conducted in an argon gas-filled glove box, in which the O_2_ and H_2_O content were maintained below 0.1 ppm.

### Characterization

An SZ680B2L microscope acquired optical images. Fourier transform infrared **(**FTIR) spectra were characterized with BRUKER VERTEX70. The morphologies of samples and elemental mapping were examined by scanning electron microscopy (SEM, Zeiss, Gemini 500) and transmission electron microscopy (TEM, Thermo Scientific, Talos F200X) equipped with energy-dispersive X-ray spectroscopy (EDX). Three-dimensional images were tested by an X-ray microscope (Zeiss, Xradia 610versa). Differential scanning calorimetry (DSC) curves were recorded using differential scanning calorimeter (DISCOVER DSC250). The crystallinity was investigated by normal X-ray diffraction (XRD, Bruker D8 Advance). X-ray photoelectron spectroscopy (XPS) spectra were collected using Thermo Fisher ESCALAB Xi^+^ using an Al Kα. The ^6^Li solid-state NMR of different gel polymers were tested by Bruker AVANCE NEO 500 MHz NMR spectrometer. The SRD results were collected at the beamline P02.1, storage ring PETRA-III, at DESY (Deutsches Elektronensynchrotron) in Hamburg, Germany.

### Electrochemical Measurements

The electrochemical stability window of CMP/VAMMT was measured using Li/SS cells at a scan rate of 1 mV s^−1^ from OCV to 6 V at room temperature. The lithium transference number for CMP/VAMMT and CMP was measured by chronoamperometry and AC impedance spectra and calculated by the following equation:

1 $${t}_{\mathrm{Li}+}=\frac{{I}_{\mathrm{s}}(\mathrm{\Delta V}-{I}_{0}{R}_{0})}{{I}_{0}(\mathrm{\Delta V}-{I}_{\mathrm{S}}{R}_{\mathrm{S}})}$$

where *I*_s_ and *I*_0_ are the steady-state and initial DC currents, respectively; *R*_s_ and *R*_0_ are the charge transfer resistances of the Li symmetric cell after and before DC polarization, respectively; and Δ*V* is 10 mV. Two lithium-metal foils were used as the non-blocking electrodes.

The ionic conductivities (*σ*) of CMP/VAMMT from 0 to 60 °C were tested by AC impedance spectroscopy (CHI-660e, CH instruments) with the electrolyte sandwiched between stainless steels, and they were calculated as follows the equation:

2 $$\sigma =\frac{\mathrm{L}}{(\mathrm{A}\times {\mathrm{R}}_{\mathrm{b}})}$$

where *L* is electrolyte thickness, *A* is the stainless-steel area and *R*_b_ is the bulk resistance of the electrolyte. Electrochemical stripping/plating for Li symmetric cells and galvanostatic cycling tests for S, LFP, NCM_523_ full cells were conducted on a Neware battery tester with galvanostatic conditions.

## Results and Discussion

### Characterizations of VAMMT and GPE/VAMMT

MMT is a layered nanoclays silicate mineral (Fig. S2) with abundant negative charges on its layers due to the surface defects [38]. These negative charges have a favorable effect on the dissociation and migration of Li^+^ in the lithium-ion batteries, as will be explained later. The schematic diagram (Fig. S3a) illustrates the synthesis of the gel polymer electrolyte and cross-linked polymer network structure (Fig. S3b). The precursor with low viscosity transforms into a cross-linked gel polymer electrolyte (CMP) under UV light (Fig. S3c). Cross-linking agent (PEGDA) enables cross-linking reaction between polymer chains, providing good mechanical strength and avoiding structural damage in VAMMT (Fig. S3d). Rheology test, nuclear magnetic resonance (^1^H NMR), FTIR and XPS can confirm the gel formation, and the conversion of monomer to polymers is almost 100% (Figs. S4-S7). It should be noted here that the intervention of the VAMMT within the gel didn't show any adverse effect on the polymer crystallization, as confirmed by the DSC analysis (Fig. S8).

**Fig. 2:**
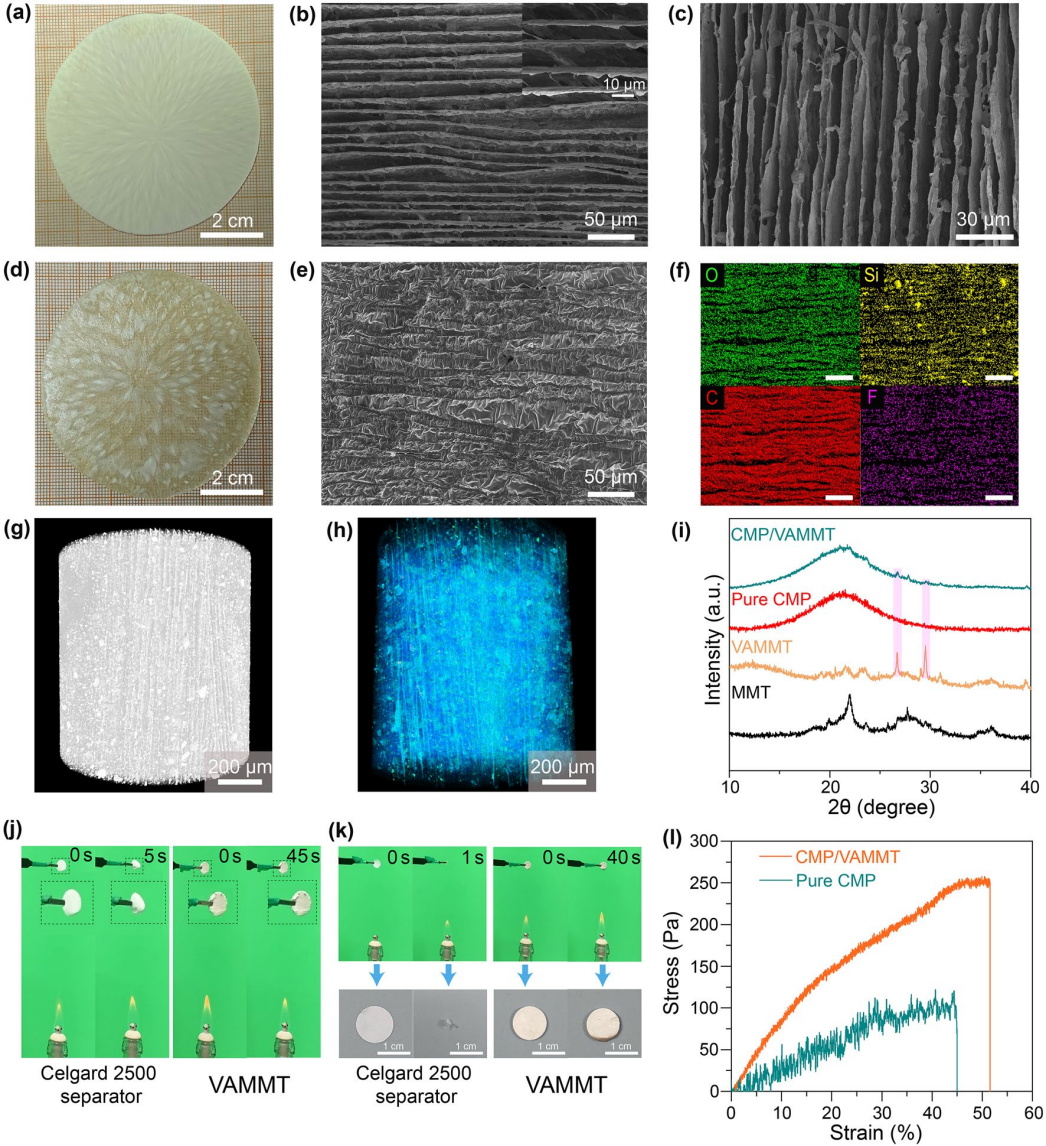
Structure characterization of VAMMT and CMP/VAMMT. **a** Digital photograph of VAMMT. **b** SEM image (from top view) of VAMMT (the inset image shows the high magnification SEM image). **c** Cross-sectional SEM image of VAMMT. **d** Digital photograph of CMP/VAMMT. **e**, **f** SEM image (from top view) of CMP/VAMMT with corresponding element mapping of O, C, Si, F. The X-ray microscope image **g** before and **h** after CMP infiltration into VAMMT (blue aera represents gel matrix); **i** XRD patterns of CMP/VAMMT and its components; digital images of flame test on VAMMT and Celgard 2500 separator **j** far from the flame and **k** close to the flame; **l** stress–strain curves of different electrolytes

Digital photographs of VAMMT (Fig. 2a) show different shades of white stripes. The light-colored areas represent MMT with wide interlayer spacing, while the darker areas represent narrower interlayer spacing. The contrast in color reflects that the ice crystals are only oriented in the vertical direction but are not confined in the plane. Further observation of the surface and cross-sectional morphology of VAMMT by SEM (Fig. 2b, c) revealed MMT nanosheets are successfully transformed into a continuous array structure with ultra-low tortuosity. After CMP infiltrates VAMMT, the features of the array structure remain intact, as can be seen from the digital photograph (Fig. 2d) and surface morphology with corresponding element mapping (Fig. 2e, f). The X-ray microscope images demonstrate that the vertical alignment of VAMMT is maintained after CMP infiltration, as shown in Fig. 2g, h (the blue area in Fig. 2h represents CMP). Further evidence of the integrity of VAMMT and their low tortuosity can be found using the internal section images of VAMMT in the X–Y plane and X–Z plane (Fig. S9). The sharp peaks in the XRD spectra of VAMMT at 26.67° and 29.52° suggest the high crystallinity of the array structure. The same peaks can be detected in the CMP/VAMMT, indicating that the layered structure of MMT is still maintained (Fig. 2i). When prepared in different sizes, no apparent changes can be observed in the VAMMT structure (Fig. S10). In terms of the mechanical properties, VAMMT increases the strength at breaking by almost 2.5 times compared with pure CMP (Fig. 2l).

Although, in general, GPEs are safer than liquid electrolytes, proper evaluation of the thermal stability is needed due to the presence of organic solvents. The TGA analysis gives a good idea about the volatile/flammable components in all the prepared materials. The TGA curves of VAMMT and CMP/VAMMT are illustrated in Fig. S11. VAMMT contains 63 wt% of MMT, which is still as high as 13.2 wt%, even in CMP/VAMMT. Practically in the combustion test (Fig. 2j, k), VAMMT shows excellent stability under the flame heat when compared to a commercial separator, which is significantly deformed.

### Electrochemical Tests of CMP/VAMMT Electrolyte

**Fig. 3:**
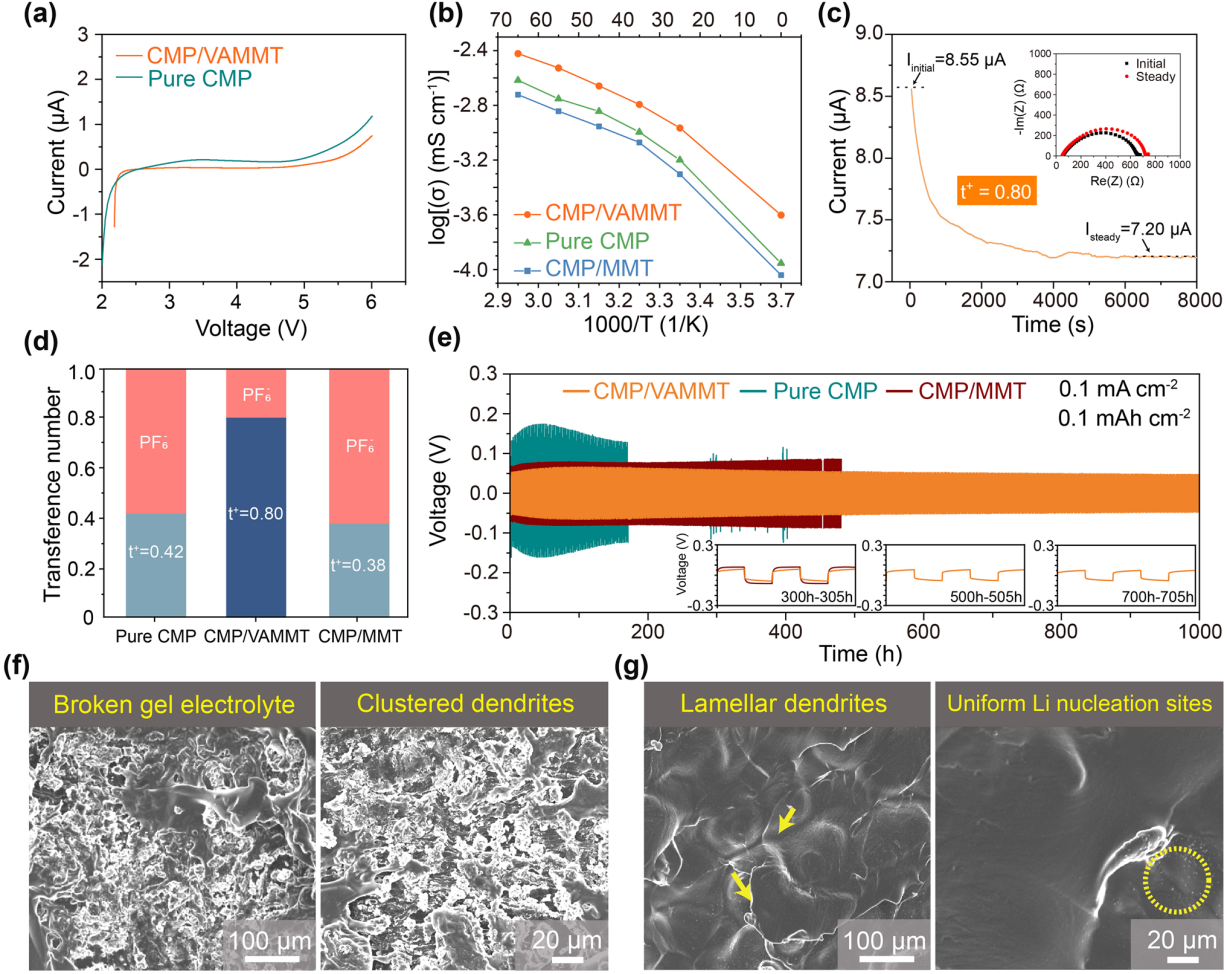
Electrochemical properties and stability of CMP/VAMMT. **a** Linear sweep voltammetry of CMP/VAMMT and the pure CMP at a sweep rate of 1 mV s^−1^. **b** Arrhenius ionic conductivity (σ) plots of CMP/VAMMT, CMP/MMT and pure CMP. **c** Current variation with time during polarization of a Li/CMP/VAMMT/Li symmetric cell (inset: the initial and steady-state AC impedance). **d** The Li^+^ transference number of different electrolytes. **e** Long-term cycling of symmetrical Li//Li cells with CMP/VAMMT, CMP/MMT and pure CMP at 30 °C (inset: voltage profile at the 300th-305th, 500th-505th and 700^th^-705^th^, respectively); SEM images of cycled Li electrodes collected from symmetrical Li//Li cells utilizing **f** pure CMP or **g** CMP/VAMMT GPEs after 20 cycles at 0.1 mA cm^−2^, 0.1 mAh cm^−2^; these tests are all carried out at 30 °C

Linear sweep voltammetry (LSV) was used to evaluate the electrochemical performance of CMP/VAMMT (Fig. 3a). The decomposition potential of CMP/VAMMT (~ 4.9 V) is higher than that of pure CMP. Then, the ionic conductivities of various electrolytes measured using blocking cells at a temperature range (0 ~ 65 °C) are illustrated in Fig. 3b. It should be noted that MMT loading in the CMP/MMT (the GPE with randomly dispersed MMT) is the same as CMP/VAMMT. The room-temperature ionic conductivity of the CMP/VAMMT is 1.08 × 10^–3^ S cm^−1^, higher than pure CMP (6.32 × 10^–4^ S cm^−1^) and CMP/MMT (4.98 × 10^–4^ S cm^−1^); the liquid electrolyte content of the three GPEs is basically the same (Fig. S12). The low conductivity of CMP/MMT is believed to be due to the aggregation of MMT at high content levels, which significantly reduces the interfaces available for Li^+^ conduction. Interestingly, CMP/VAMMT maintains high ionic conductivity of 2.49 × 10^–4^ S cm^−1^ at 0 °C. As shown in Fig. 3c, we further measured the Li^+^ transference number (t^+^) of CMP/VAMMT using Li symmetric cell. Figure 3d displays that the t^+^ is boosted from 0.42 to 0.80 by introducing VAMMT. The t^+^ of CMP/MMT is only 0.38, lower than that of pristine CMP, further proving the role of the MMT aggregation in hindering the ions transport (Fig. S13). The construction of continuously aligned interfaces improves the continuity of Li^+^ migration and reduces the migration barrier of Li^+^ between different phases [39]. Hence, the t^+^ of CMP/VAMMT is effectively enhanced. To observe the interfacial stability during Li stripping/plating, symmetrical Li cells with different GPE were tested at 30 °C. Under 0.1 mA cm^−2^, the cell using CMP electrolyte exhibits unstable voltage lasting a short time (170 h) owing to the poor mechanical stability and uneven diffusion paths of Li^+^ (Fig. 3e). Introducing MMT to the electrolyte, even if randomly oriented, improved the cell stability with low voltage hysteresis that lasted more than 500 h. This may be because MMT weakens the binding of Li^+^ by polymer chains through surface interaction, thereby reducing the migration barrier of Li^+^ from the polymer to the electrode surface [32]. The rapid and even diffusion of Li^+^ along VAMMT enables highly stable cyclic performance for over 1000 h with a very low and decreasing voltage hysteresis with time. To further investigate the tolerance of GPEs at high current densities, we first performed critical current density (CCD) tests on CMP/VAMMT and pure CMP in Li symmetric cells (Fig. S14a). The polarization potential of CMP/VAMMT is smaller than that of pure CMP at different current densities, and CMP/VAMMT also shows higher critical current density of 0.25 mA cm^−2^ 0.25 mAh cm^−2^. Meanwhile CMP/VAMMT also showed stable cycling performance at higher current density (> 300 h) and CCD (> 100 h) (Fig. S14b-c).

The EIS Nyquist plots of Li/GPE/Li symmetrical cells were measured to investigate the interfacial compatibility between different GPEs and the Li electrode. It can be seen in Fig. S15 that the Li/CMP/VAMMT/Li cell possessed the low interface resistance of 326 Ω, much smaller than 669 Ω of the Li/pure CMP/Li cell. The reason can be ascribed that the supporting property and the neat array structure of VAMMT ensure good interfacial contact between CMP/VAMMT and Li metal. In addition, after 5 and 50 cycles, the impedances of the Li/CMP/VAMMT/Li cell and Li/pure CMP/Li cell are 475, 1247 Ω and 414, 1758 Ω, respectively. Irregular dendrite growth and uneven ion migration lead to severe deterioration of the internal interface of Li/CMP/Li cells, while VAMMT can induce uniform migration of Li^+^ at the electrode to form a stable interface that exhibits lower contact resistance.

It is worth mentioning that messy clusters of Li dendrites can be seen on the surface of the Li electrode using pristine CMP after 20 cycles (Fig. 3f), while the surface of the Li electrode utilizing CMP/VAMMT is relatively flat and smooth (Fig. 3g). Moreover, CMP/VAMMT membrane did not show signs of damage or deformation after cycling and can be removed completely from Li electrodes (Fig. S16). It can be concluded that continuous interfaces in VAMMT provide a new route for Li^+^ migration and weaken the dependence of Li^+^ on free solvents in GPEs, thereby promoting better electrochemical stability of GPEs.

### Understanding Li^+^ Transport Machnism in CMP/VAMMT

**Fig. 4:**
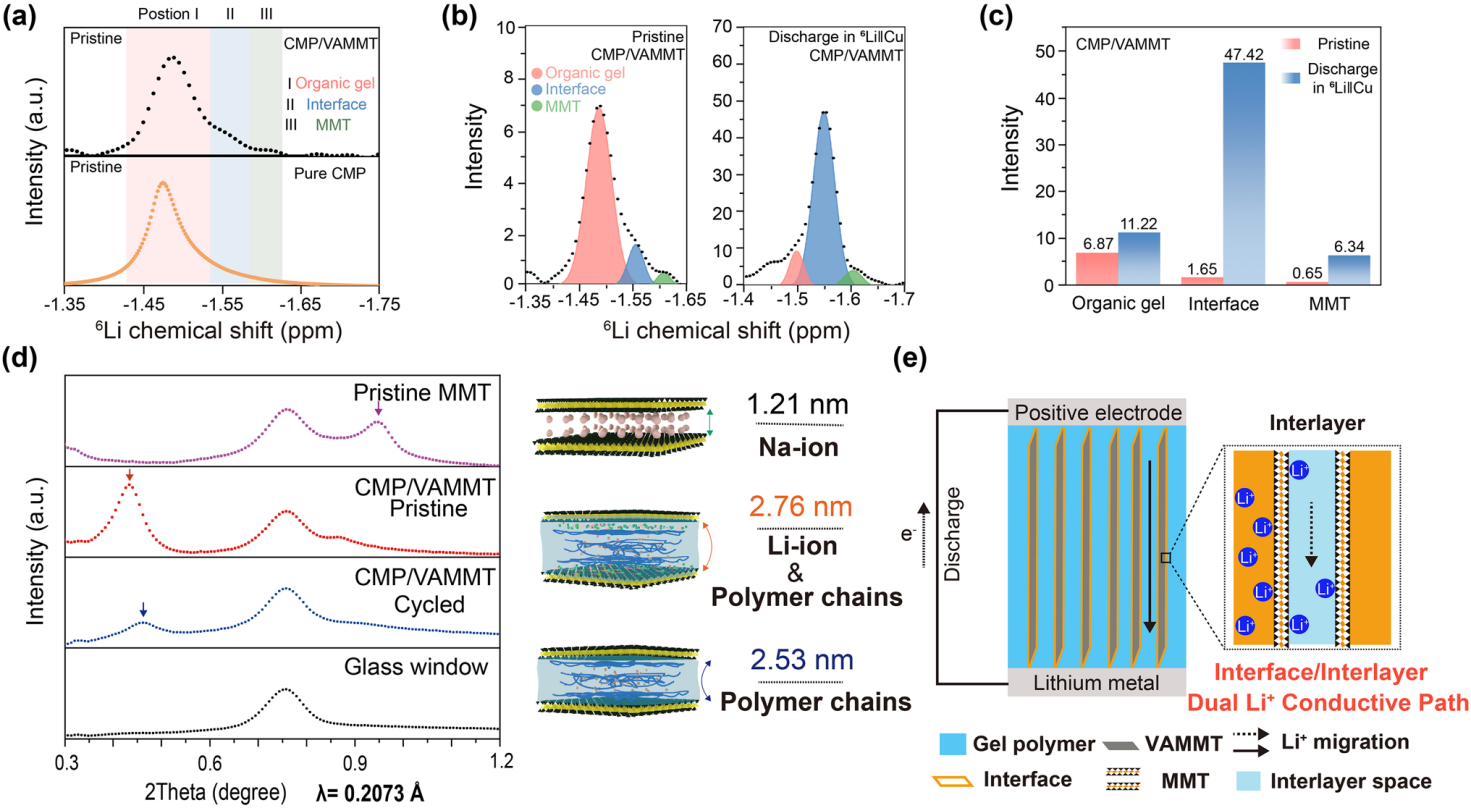
Understanding the Li^+^ migration mechanism in CMP/VAMMT. **a** ^6^Li SSNMR spectra of pure CMP and CMP/VAMMT in the pristine state (the three-color zones represent the different chemical environments of Li ion in CMP/VAMMT, whereas in pure CMP only the chemical environments represented by red are present). **b** ^6^Li SSNMR spectra of CMP/VAMMT before (left) and after (right) discharge in the ^6^Li‖Cu cell. **c** Comparison of changes in different peak intensities in the Fig. 4b. **d** Synchrotron radiation X-ray diffraction (λ = 0.2073 Å) patterns of pristine MMT, pristine CMP/VAMMT, cycled CMP/VAMMT and the glass window (use for testing) (right: Schematics illustrating the layer spacing changes of MMT layers in pristine MMT, pristine CMP/VAMMT, cycled CMP/VAMMT from the result of the SRD pattern). **e** Schematic diagram of dual Li^+^ migration path in the CMP/VAMMT

To shed more light on the mechanism of Li^+^ transport in CMP/VAMMT, we used ^6^Li SSNMR and SRD. ^6^Li SSNMR is conducted coupled with ^6^Li/^7^Li isotope-replacement method with ^6^Li metal as lithium source in the ^6^Li‖Cu cell [40, 41]. ^6^Li^+^ is stripped from one ^6^Li-metal electrode and plated on the other, going through the composite GPE. ^6^Li SSNMR is firstly recorded for the as-prepared, i.e., before stripping/plating, CMP and CMP/VAMMT to distinguish different local environments of ^6^Li^+^ (Fig. 4a). ^6^Li resonance of the pure CMP is at −1.49 ppm, which corresponds to the Li^+^ distributed around the polymer chains and solvent. The ^6^Li spectrum recorded for the fresh CMP/VAMMT shows additional two peaks at −1.56 ppm and −1.60 ppm, corresponding to the Li^+^ at the polymer-VAMMT interface and on the VAMMT surface, respectively. After ^6^Li||CMP/VAMMT||Cu cell was discharged at 50 μA cm^−2^ for 2 h, the intensity of the peak corresponding to Li^+^ in the organic phase significantly reduced, while that for Li^+^ at the polymer-VAMMT interface dominates the spectrum (Fig. 4b). Similar results can also be obtained from the ^6^Li SSNMR before and after discharging through ^7^Li||CMP/VAMMT||Cu cells (Fig. S17). The significant peak intensity increases in Li^+^ at the interface (2774% growth, Fig. 4c), which confirms that the Li^+^ migration along the polymer-VAMMT interface is the mean reason for the high ionic conductivity of CMP/VAMMT. Also, the increased intensity (0.65 to 6.34) of the peak at -1.60 ppm suggests a significant role of the Li^+^ migration within the VAMMT in improving the overall ionic conductivity of the CMP/VAMMT gel electrolyte.

Since MMT is in the form of two-dimensional nanosheets, the movement of Li^+^ on the MMT interlayer might change its layer spacing. However, the XRD of ordinary light sources cannot distinguish these kinds of small changes [41, 42]. Thus, we used the high-resolution SRD of the CMP/VAMMT to measure the interlayer space of MMT before and after cycling 5 times of charge and discharge at 0.1 mA cm^−2^ in the symmetrical Li||Li cells. From Fig. 4d, the diffraction angles of pristine MMT (P-MMT), fresh CMP/VAMMT and CMP/VAMMT after cycling are 0.948°, 0.432° and 0.461°, respectively. According to the Bragg equation, the interlayer space in the P-MMT is 1.21 nm [30]. The interlayers of MMT filled with polymer chains and solvated Li^+^ decrease from 2.76 to 2.53 nm after cycling, indicating that some of the Li^+^ migration within MMT layers has been shifted to migrate at the polymer-VAMMT interface. Therefore, the vertical-aligned MMT interlayers in the VAMMT not only create a low-tortuosity Li^+^ migration interface but also form a continuous path inside the MMT interlayers, further improving the ionic conductivity (Fig. 4e). In addition, this interaction between VAMMT surfaces and Li^+^ can be further proved by XPS spectra (details in Fig. S18).

**Fig. 5:**
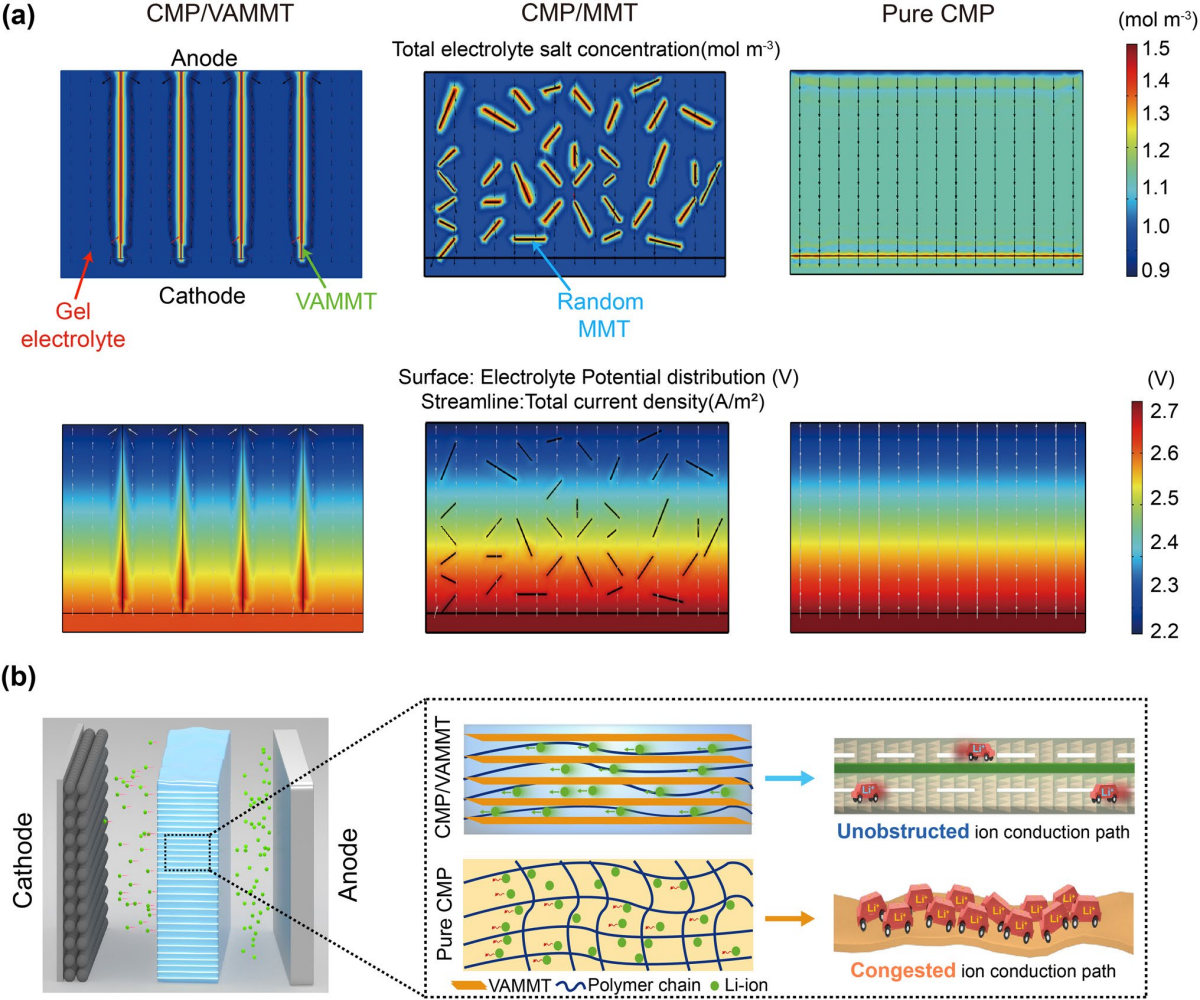
Li^+^ migration visual analysis. **a** Modeling total electrolyte salt concentration for the CMP/VAMMT, CMP/MMT, pure CMP (top) and corresponding mapping of electrolyte potential distribution (bottom). **b** Schematic diagram of CMP/VAMMT in the cell (left) and comparison of Li.^+^ transport between CMP/VAMMT and pure CMP (right)

We used 'COMSOL Multiphysics' (COMSOL) numerical analysis of the current distribution in different GPEs (CMP/VAMMT, CMP/MMT, CMP) to visualize the Li^+^ diffusion process. As shown in Fig. 5a (top), gel electrolyte and MMT are distinguished by the different colors, which change from blue to red with the increase in the electrolyte salt concentration. The red lines represent the Li^+^ mainly concentrated on the VAMMT layers, in line with the experimental results. The electrolyte potential also changes along with the VAMMT layers in Fig. 5a (bottom). Although the electrolyte salt concentrates around the MMT nanosheets in the CMP/MMT, there are no obvious changes in the electrolyte potential mapping of the CMP/MMT, which makes the diffusion of Li^+^ still limited by the continuous phase of polymer chains and solvent. It is obvious that low tortuous VAMMT allows for uniform and fast transport of Li^+^. All in all, VAMMT can be visualized as a countless well-distribute ion transport highway, ensuring fast and uniform ion migration (Fig. 5b).

### Battery Performance Using CMP/VAMMT Electrolyte at Wide Operating Temperature

**Fig. 6:**
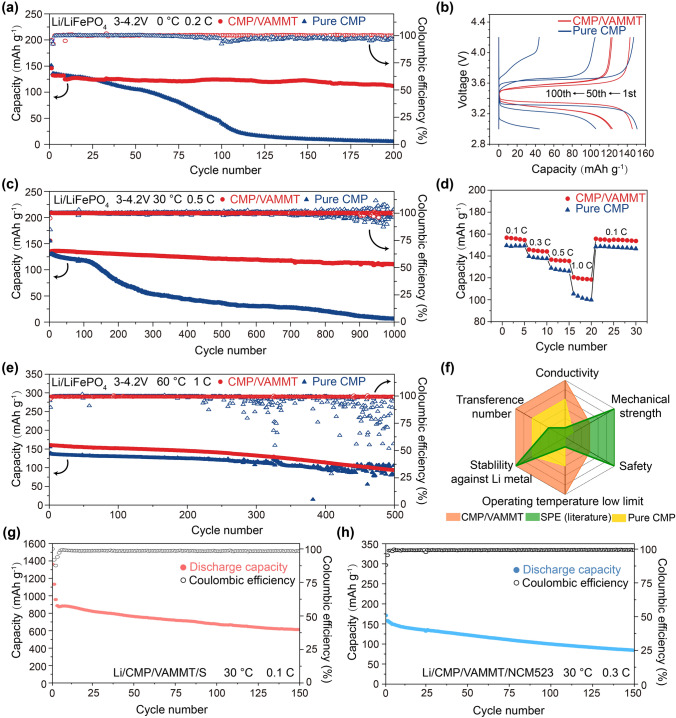
Cycling performance of CMP/VAMMT in full cells. **a** Long cycle performance of Li/CMP/VAMMT/LFP cells at 0.2C, 0 °C. **b** Corresponding voltage profile at different cycles in a). **c** Long cycle performance of Li/CMP/VAMMT/LFP cells at 0.5C, 30 °C. **d** Cycling performance of Li/CMP/VAMMT/LFP cell at different charging rates, cycled at 30 °C. **e** Long cycle performance of Li/CMP/VAMMT/LFP cells at 1C, 60 °C. **f** The performance of the CMP/VAMMT electrolyte compared with the pure CMP and SPEs reported in the literature. Cycle performance of **g** Li/CMP/VAMMT/S cells at 0.1C and **h** Li/CMP/VAMMT/NCM523 cells at 0.3C, 30 °C

To evaluate the cycling and rate performance of CMP/VAMMT, coin cells were assembled with LFP cathodes and Li metal as the anodes (Fig. S19). We also tested controlled cells with pure CMP for comparison. The batteries using GPEs often suffer rapid deterioration at low operating temperatures due to the poor ionic conductivity and the rapid growth of lithium dendrites. However, when testing the battery with CMP/VAMMT at 0 °C, a stable cycling performance is observed for 200 cycles at 0.2C (Fig. 6a) with a low-voltage plateau (Fig. 6b). In comparison, the cell with CMP electrolyte decays dramatically within 75 cycles. The rate performance of Li/CMP/VAMMT/LFP cell was also performed at Fig. S20. Even when the current density is increased to 0.3C, the cell still maintains a high capacity. VAMMT also maintains uniform Li^+^ conduction, and the cell demonstrates excellent stability at 0.1C (Fig. S21). At 30 °C, the CMP/VAMMT achieved 1000 cycles with the capacity retention of 85.8% at 0.5C in Fig. 6c. The capacity of the Li||pure CMP||LFP decreases sharply after the first 100 cycles, reaching 5.8 mAh g^−1^ after 1000 cycles with an average retention of 4.5%. The cells with CMP/VAMMT also show better capacity retention at 0.3C and 1C (Fig. S22), even at a high rate of 2C (Fig. S23). The impedance of CMP/VAMMT is still much smaller than that of pure CMP after cycling (Fig. S24), indicating that VAMMT significantly improves Li^+^ transport capacity and reduces the occurrence of side reactions. An excellent rate performance could be achieved when CMP/VAMMT is used, as shown by the cycling tests at 30 °C in Fig. 6d. The cells cycled at 0.1C, 0.3C, 0.5C and 1C; the Li||CMP/VAMMT||LFP cell delivers high specific capacities of 157, 146, 137 and 121 mAh g^−1^, respectively. When cycling back at 0.1C the Li||CMP/VAMMT||LFP cell could still deliver 157 mAh g^−1^, indicating superior rate performance. We also tested the CMP/VAMMT in cells at high temperatures to examine any deterioration in the cell due to gel deformation or internal solvent volatilization. Compared with the sharply fluctuating Coulombic efficiency in the Li||pure CMP||LFP cell after 200 cycles at 60 °C, the Li||CMP/VAMMT||LFP cells demonstrated a stable coulombic efficiency of around 99.4% for more than 500 cycles (Fig. 6e). Furthermore, when matched with S or NCM523 cathodes (Fig. 6g, h), the cells with CMP/VAMMT electrolyte can achieve good cyclic performance and stable voltage plateau (Figs. S25 and S26). Pouch-type LFP/CMP/VAMMT/Li cell was further made to demonstrate the application of the CMP/VAMMT for totally sate lithium batteries. As shown in Fig. S27, the pouch cell can perform charge–discharge cycles normally and can still work normally after folding and cutting, indicating that CMP/VAMMT has good safety and practical application potential. Therefore, VAMMT that introduces continuous interface/interlayer dual-ion-conducting paths shows a unique set of properties, including high conductivity, electrochemical stability, excellent capacity retention at low and high temperatures, good mechanical properties and improved safety aspects (Fig. 6f, details in Table S1). The new concept of designing GPEs provides a new pathway toward all-weather practical solid-state batteries (Fig. S28 and Table S2).

## Conclusions

This paper introduced a new concept for GPE based on using VAMMT as ICA impregnated with CMP for SSLMBs. Introducing VAMMT to the GPE enhanced its ionic conductivity (1.08 mS cm^−1^), transference number (0.80) and electrochemical window (> 4.9 V), as well as thermal stability and mechanical flexibility. In addition, we discovered that the mechanism of fast Li^+^ migration is interface/interlayer dual Li^+^-conduction pathways in the composite GPE by the ^6^Li SSNMR, SRD and simulations. Therefore, when CMP/VAMMT was used in SSLMBs with different cathodes like sulfur, NCM523, LFP, the cells demonstrated outstanding rate performance and cycling stability at a wide temperature range from 0 to 60 °C in the Li/LFP cells. For example, the Li/CMP/VAMMT/LFP maintained 85.6% of its original capacity after 1000 cycles at 30 °C. Despite the excellent performance of GPE/VAMMT, there is still plenty of room for further improvement by changing or introducing different polymers and specific additives. Thus, we believe this approach has the potential to achieve all-weather practical solid-state batteries under a broad range of conditions.

## Supplementary Information

Below is the link to the electronic supplementary material.

Supplementary file1 (PDF 2100 kb)
