# Supplementary material for: Quasi-Solid-State Ion-Conducting Arrays Composite Electrolytes with Fast Ion Transport Vertical-Aligned Interfaces for All-Weather Practical Lithium-Metal Batteries
Source: Nanomicro Lett. 2022 Oct 31;14:210. doi: 10.1007/s40820-022-00952-z (PMC9622961; doi:10.1007/s40820-022-00952-z)
Supplement: Supplementary file 1 — Supplementary file1 (PDF 2100 kb) [file 40820_2022_952_MOESM1_ESM.pdf]

Supporting Information for

# Quasi-Solid-State Ion-Conducting Arrays Composite Electrolytes with Fast Ion Transport Vertical-Aligned Interfaces for All-Weather Practical Lithium-Metal Batteries

Xinyang Li[1], Yong Wang[1, 2], Kai Xi[1], Wei Yu[1], Jie Feng[1], Guoxin Gao[1], Hu Wu[1], Qiu Jiang[3, 4], Amr Abdelkader[5], Weibo Hua[6, 7], Guiming Zhong[8], Shujiang Ding[1, *]

[1]School of Chemistry, Xi'an Key Laboratory of Sustainable Energy Materials Chemistry, State key laboratory of Electrical Insulation and Power Equipment, Xi'an Jiaotong University, Xi'an 710049, P. R. China

[2]State Key Laboratory for Mechanical Behaviour of Materials, Xi'an Jiaotong University, Xi'an 710049, P. R. China

[3]Yangtze Delta Region Institute (Huzhou), University of Electronic Science and Technology of China, Huzhou, Zhejiang 313001, P. R. China

[4]University of Electronic Science and Technology of China, School of Materials and Energy, Chengdu 610054, P. R. China

[5]Faculty of Science and Technology, Bournemouth University, Talbot Campus, Fern Barrow, Poole, BH12 5BB, United Kingdom

[6]Institute for Applied Materials-Energy Storage Systems (IAM-ESS), Karlsruhe Institute of Technology (KIT), 76344, Eggenstein-Leopoldshafen, Germany

[7]School of Chemical Engineering and Technology, Xi'an Jiaotong University, Xi'an, Shaanxi, 710049, P. R. China

[8]Laboratory of Advanced Spectroelectrochemsitry and Liion Batteries, Dalian Institute of Chemical Physices, Chineses Academy of Sciences, Dalian, 116023, P. R. China

*Corresponding author. E-mail: dingsj@mail.xjtu.edu.cn (Shujiang Ding)

## Supplementary Figures and Tables

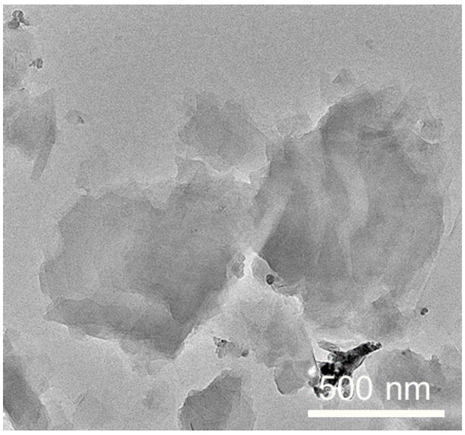


**Fig. S1** TEM images of MMT

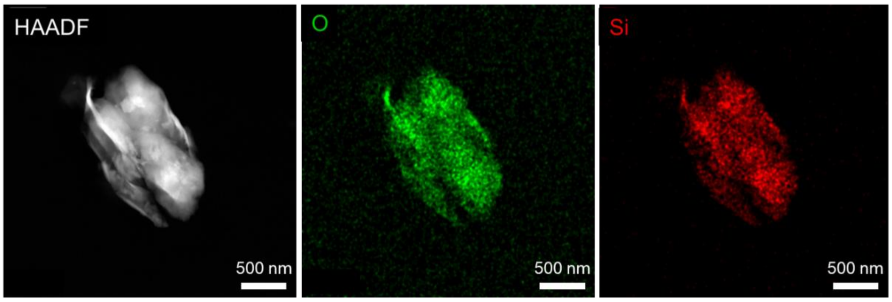


**Fig. S2** HADDF-STEM images of MMT

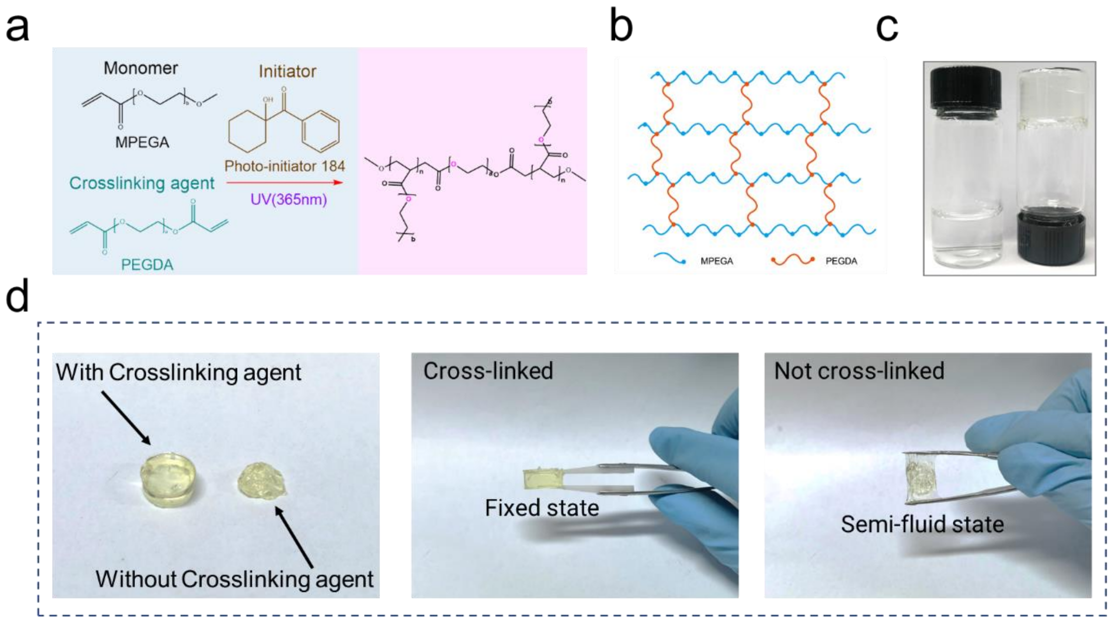


**Fig. S3** The cross-linking process of gel polymer (**a**) with schematic diagram (**b**). The digital photo of liquid precursor(left) and gel polymer(right) (**c**). Comparison of digital photos of gel polymer before and after adding crosslinking agent (**d**)

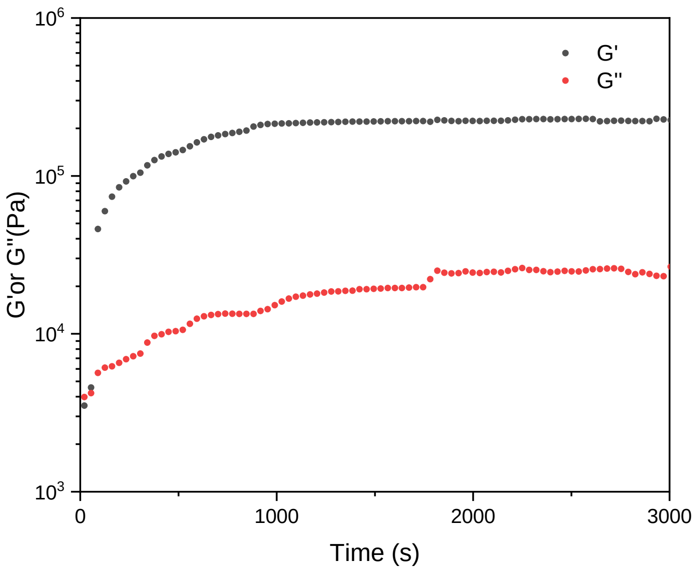


**Fig. S4** Variation of storage modulus (G') and loss modulus (G'') with polymerization time

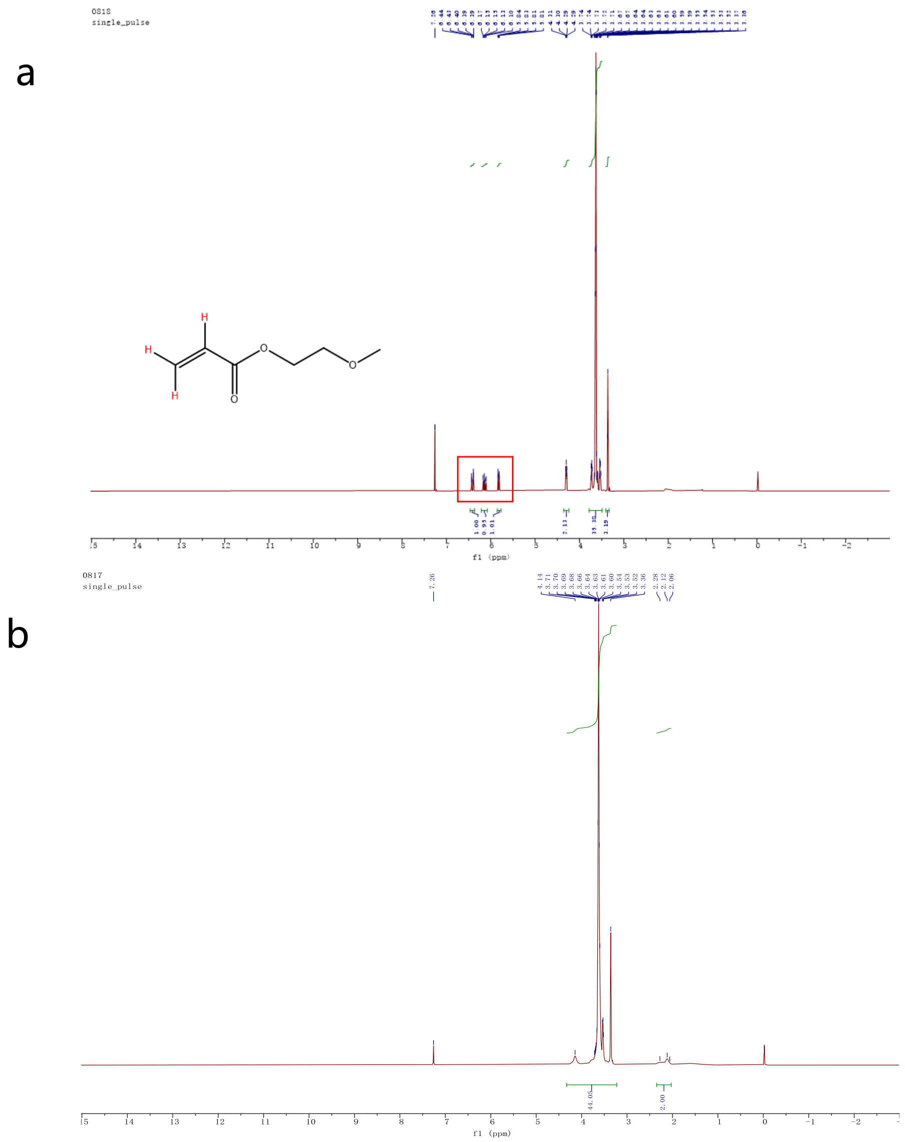


**Fig. S5** $^1$H NMR spectra of monomers and polymer. (**a**) Before polymerization, (**b**) After polymerization

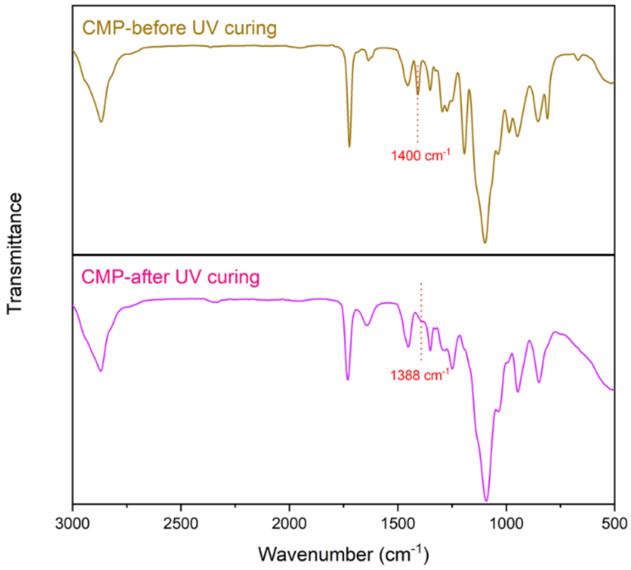


**Fig. S6** The Fourier transform infrared (FT-IR) transmittance spectra of CMP before (top) and after (bottom) UV curing

The change in the Fourier transform infrared (FTIR) transmittance spectra before and after polymerization correspond the shift of $–CH_3$ vibration peak (from 1400 $cm^{-1}$ to 1388 $cm^{-1}$).

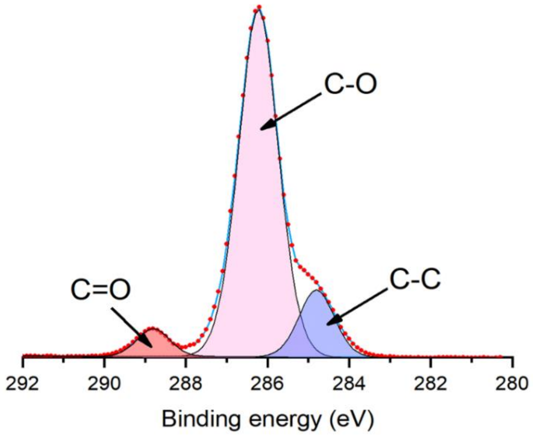


**Fig. S7** C 1s X-ray photoelectron spectroscopy (XPS) spectrum of CMP after UV. The absence of the C=C peak after curing indicates successful polymerization

The disappear of C=C peak in the C 1*s* X-ray photoelectron spectroscopy (XPS) spectra of CMP indicates the progress of the polymerization.

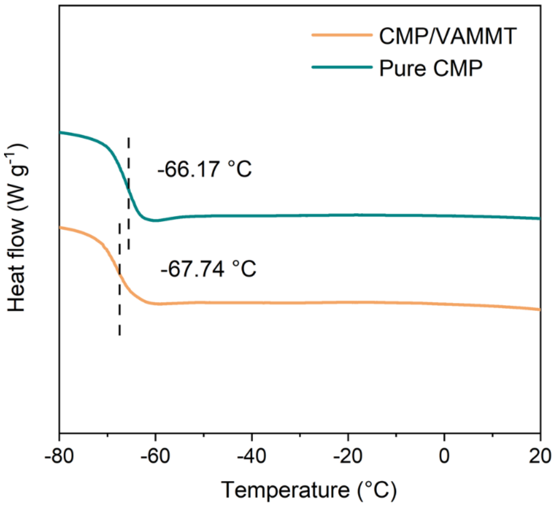


**Fig. S8** DSC curves of CMP/VAMMT and pure CMP

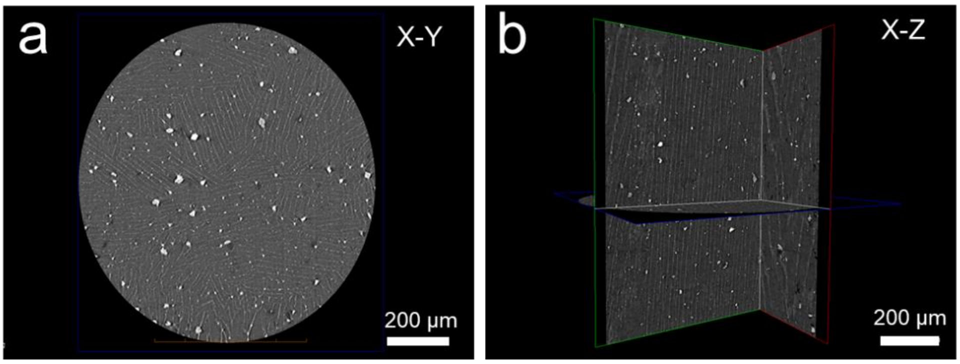


**Fig. S9** the internal sectional images of VAMMT in the X-Y plane (**a**) and X-Z plane (**b**) exhibit vertical array-like structure of VAMMT

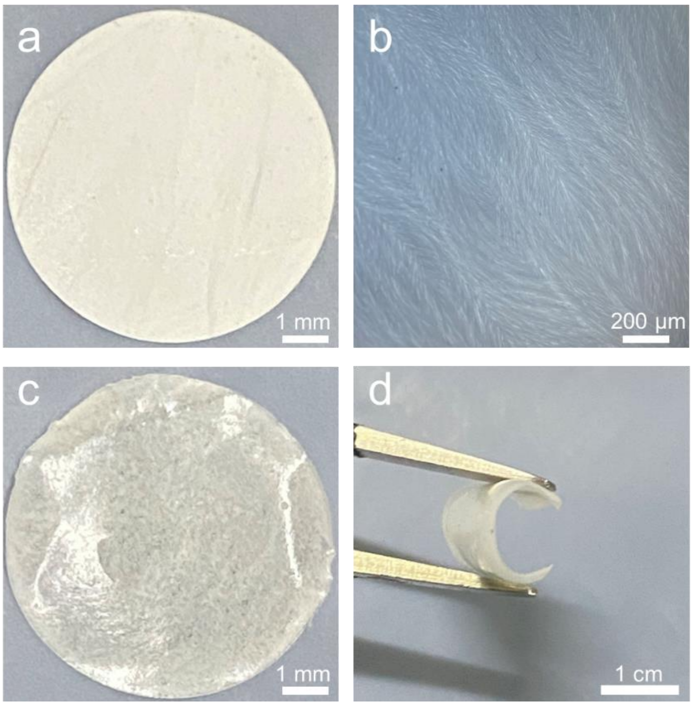


**Fig. S10** Digital image of small-size VAMMT (**a**) with an optical image (**b**) and small-size CMP/VAMMT (**c**) with a demonstration of the flexibility (**d**)

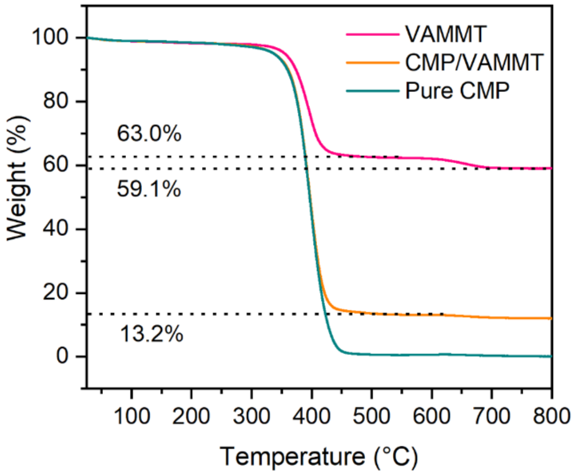


**Fig. S11** TGA curves of CMP/VAMMT and pure CMP

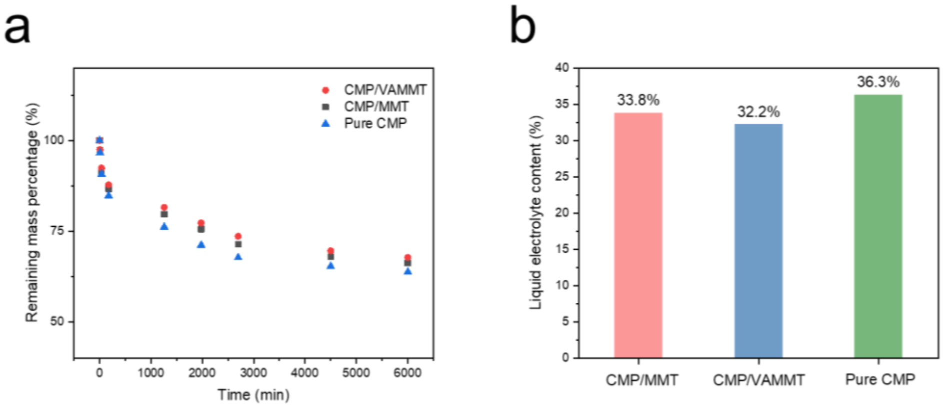


**Fig. S12** Changes in the quality of different GPEs over time in an oven at 80°C (**a**). Content of liquid electrolyte in different GPEs (**b**)

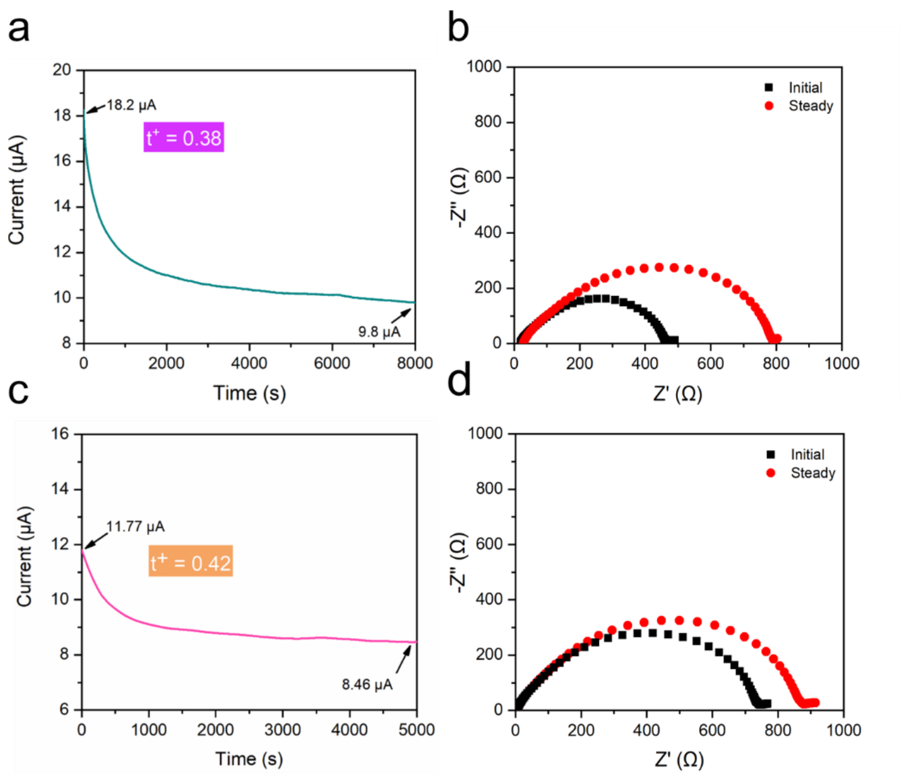


**Fig. S13** Current variation with time during polarization of Li/CMP/MMT/Li (**a**) with the initial and steady-state AC impedance (**b**) and Li/Pure CMP/Li (**c**) with the initial and steady-state AC impedance of the cell (**d**)

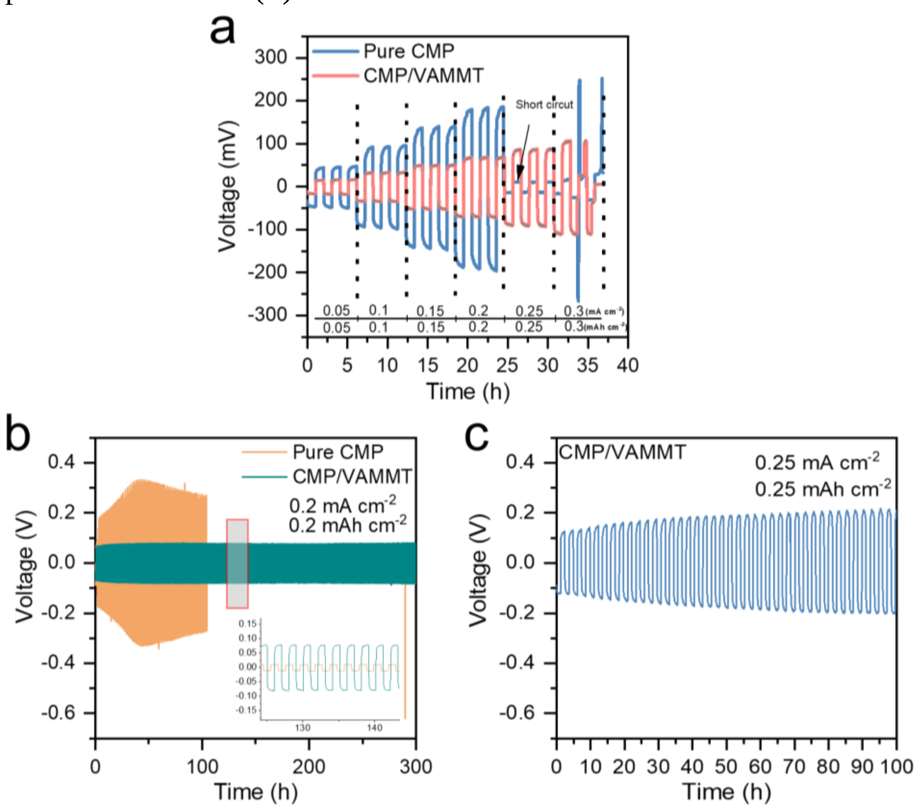


**Fig. S14** Critical current density tests on CMP/VAMMT and Pure CMP in symmetric cell, each cell was cycled three times at specific current density (**a**). Long-term cycling of symmetrical Li//Li cells with CMP/VAMMT and pure CMP at 0.2 mA cm$^{-2}$, the lithium plating capacity is 0.2 mAh cm$^{-2}$ per cycle (**b**). The cycle performance of Li/CMP/VAMMT/Li at critical current density (0.25 mA cm$^{-2}$, 0.25 mAh cm$^{-2}$) (**c**)

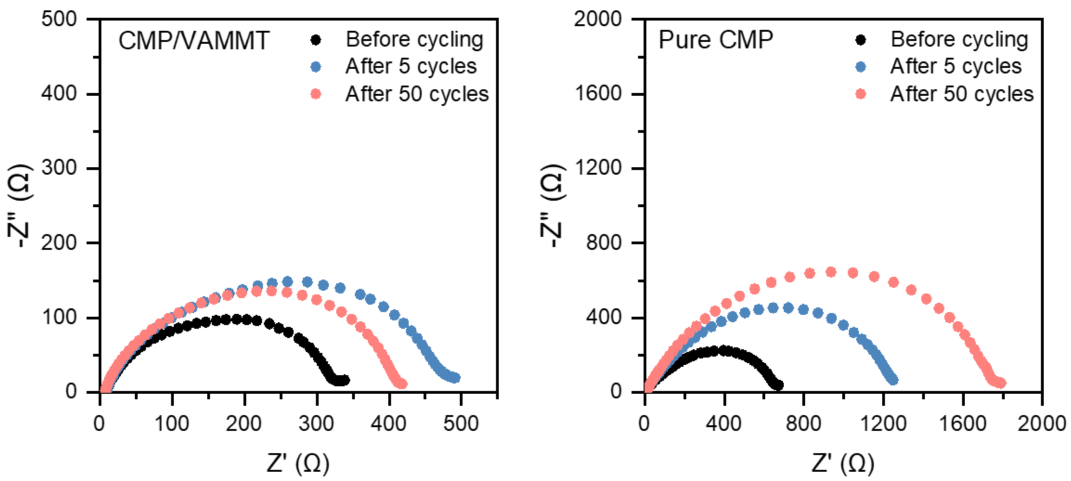


**Fig. S15** The EIS Nyquist plots of Li/GPE/Li symmetrical cells

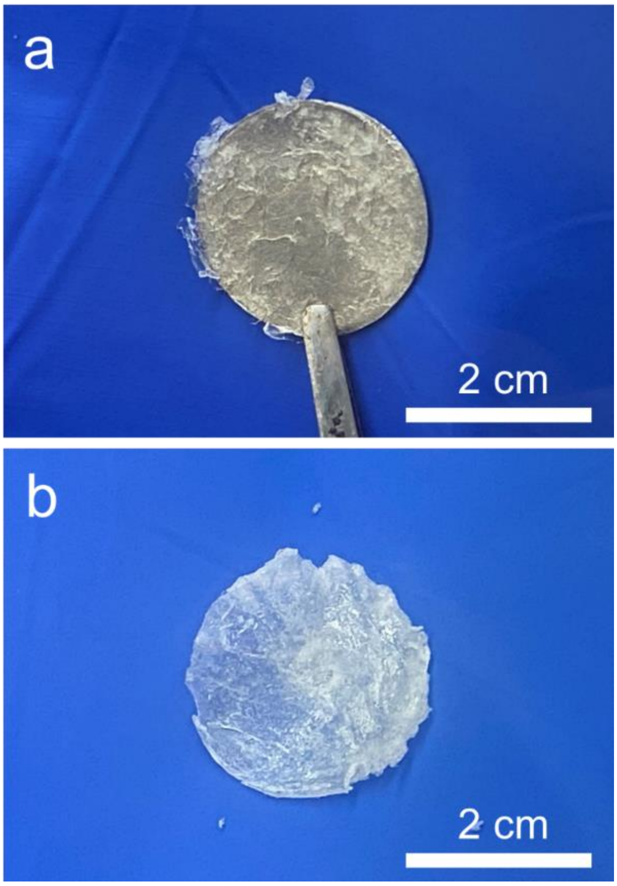


**Fig. S16** Optical photo of Pure CMP with Li anode (**a**) and CMP/VAMMT membrane (**b**) in symmetrical Li cells after the 20th cycle (corresponding to Fig. 4g-h)

The images show that pure CMP electrolyte has poor mechanical strength and cannot be removed from the cell. CMP/VAMMT, on the other hand, has good mechanical strength. It is also clear that CMP/VAMMT has smooth surface with no obvious dendrite or cracks, which can be attributed to the uniform distribution of Li-ion on the surface of the Li anode.

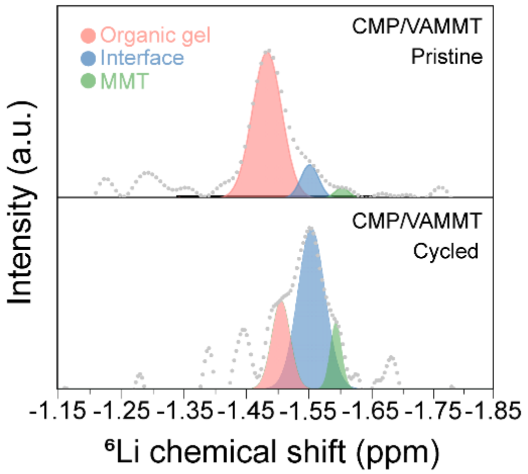


**Fig. S17** The $^{6}Li$ SSNMR spectra of CMP/VAMMT before and after cycling in the $^{7}Li$‖Cu cell

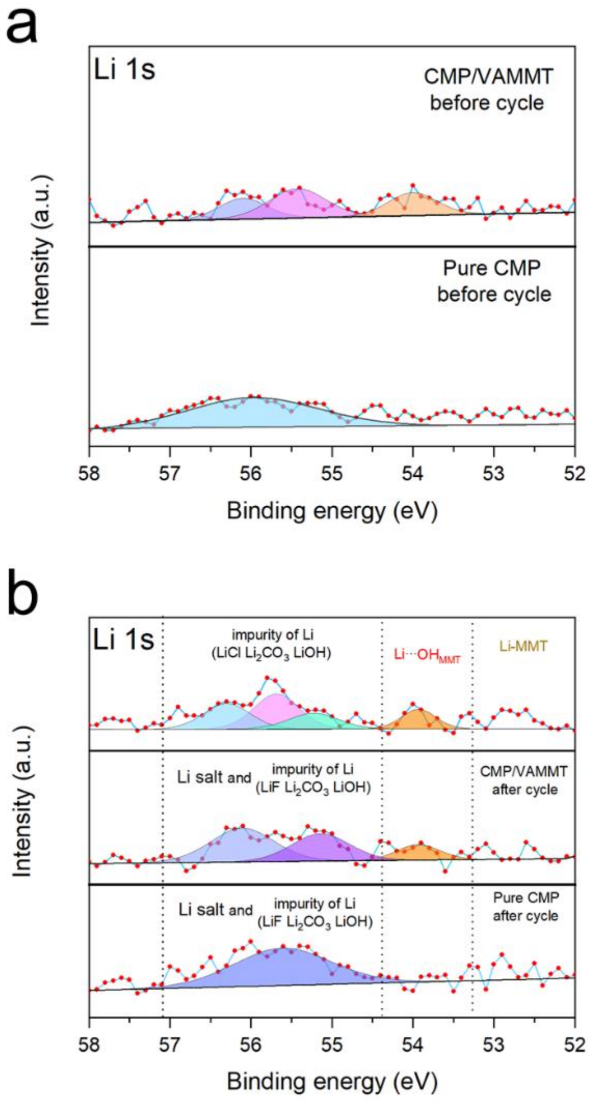


**Fig. S18** Li 1S X-ray photoelectron spectroscopy (XPS) spectra of CMP and CMP/VAMMT before (**a**) and after (**b**) cycling

In order to investigate the interaction between Li-ions and the surface of MMT, we tested CMP and CMP/VAMMT before and after cycling. However, the signal is very weak since a trace

amount of lithium is on the surface of the gel electrolyte, and the challenge to excite light elements by ordinary x-rays. Only one broad peak appears in the XPS spectra of CMP before cycling, while three different peaks appear in the XPS spectra of CMP/VAMMT. This result shows that VAMMT does have an unknown effect on Li-ions in the gel electrolyte. Therefore, we prepared Li-MMT according to the literature to further determine what the peaks represent. The XPS spectra of Li-MMT is illustrated in Supplementary Fig. 16(b). The results obtained in this way are inaccurate because XPS can only get information on the sample's surface. However, combined with $^{6}$Li solid-state NMR results, we can conclude that Li-ions interact with VAMMT in the CMP/VAMMT.

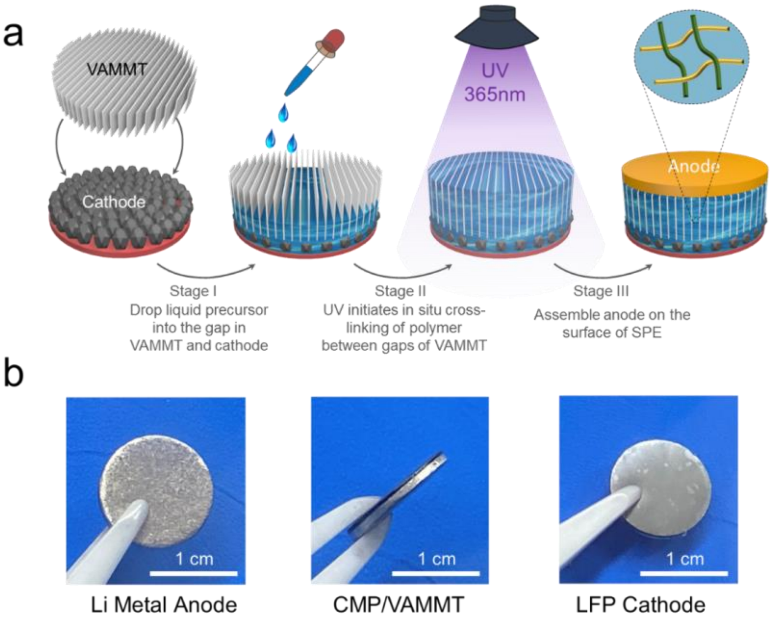


**Fig. S19** Schematic diagram of the full cell assembly process (**a**) and digital photos of the LFP cell with CMP/VAMMT (**b**)

Gel electrolytes have certain advantages in terms of their preparation compared with all solid-state electrolytes; especially in-situ polymerization can be used in the preparation method. In-situ polymerization includes UV initiation, thermal initiation, or non-covalent cross-linking, thus avoiding the use of large amounts of solvent and reducing pollution to the environment. Moreover, Interface problems like electrode-electrolyte interface and framework-electrolyte interface can be effectively solved. However, complex interface problems often mask the material's advantages, which is an important reason we use gel electrolytes to study vertical-aligned materials in the lithium metal battery.

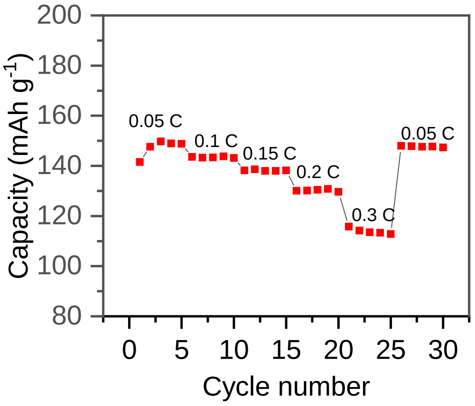


**Fig. S20** Rate performance of Li/CMP/VAMMT/LFP at 0 °C

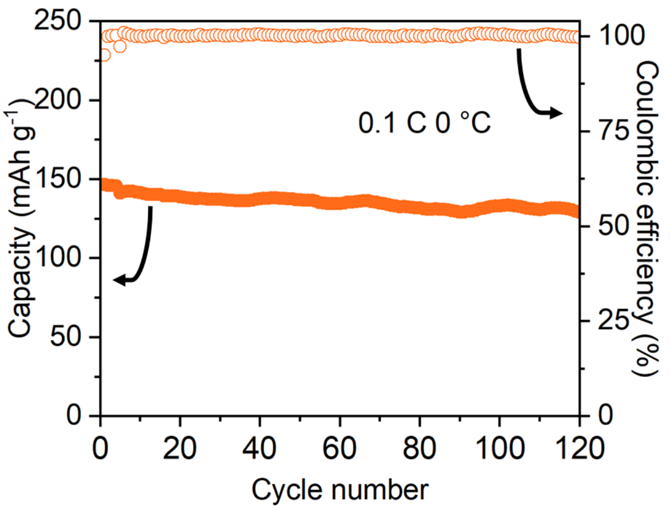


**Fig. S21** Long cycle performance of CMP/VAMMT at 0.1 C, 0°C in the Li/LFP cells

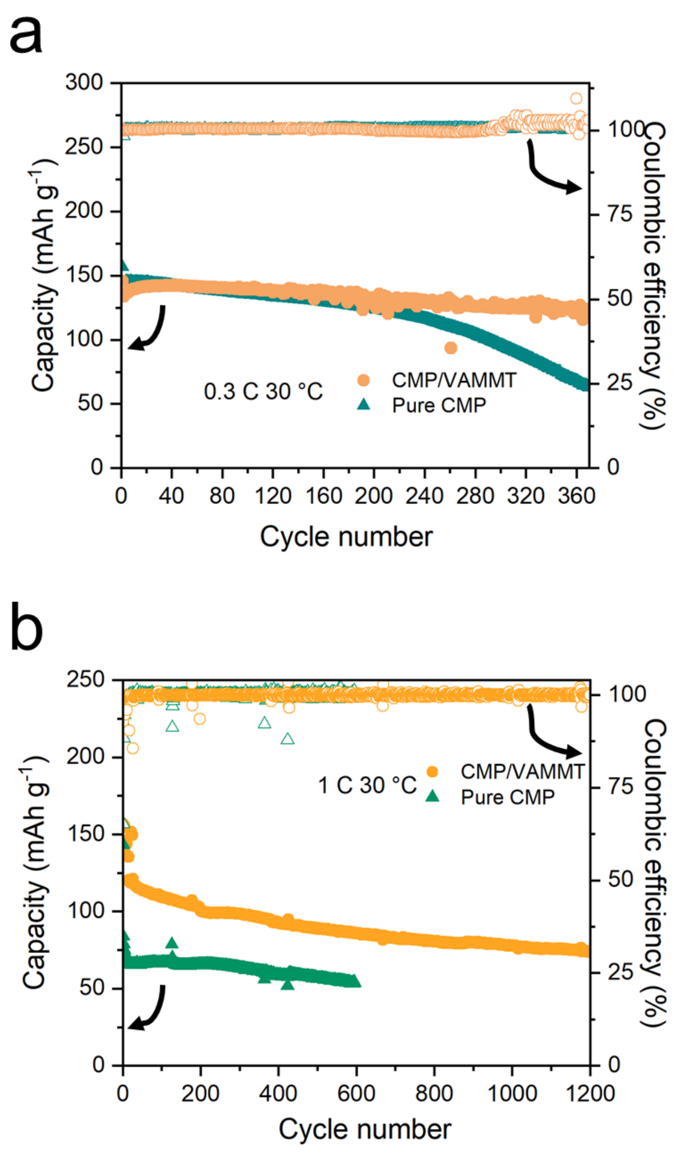


**Fig. S22** Long cycle performance of CMP/VAMMT and pure CMP at 0.3 C, 30°C (**a**); 1 C, 30°C (**b**) in the Li/LFP cells

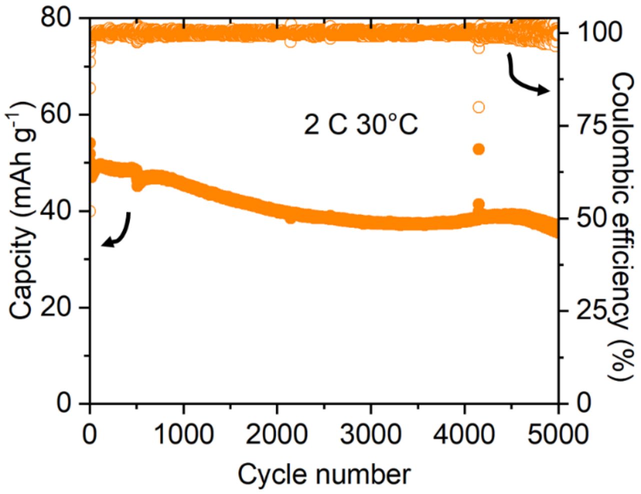


**Fig. S23** Long cycle performance of CMP/VAMMT at 2 C, 30°C in the Li/LFP cells

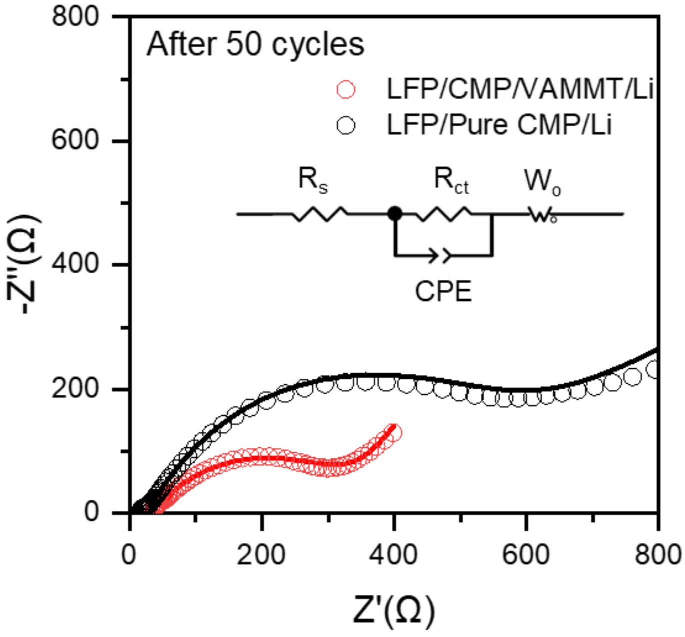


**Fig. S24** EIS curve of LFP/CMP/VAMMT/Li and LFP/pure CMP/Li cells after 50 cycles at 0.5 C, 30°C

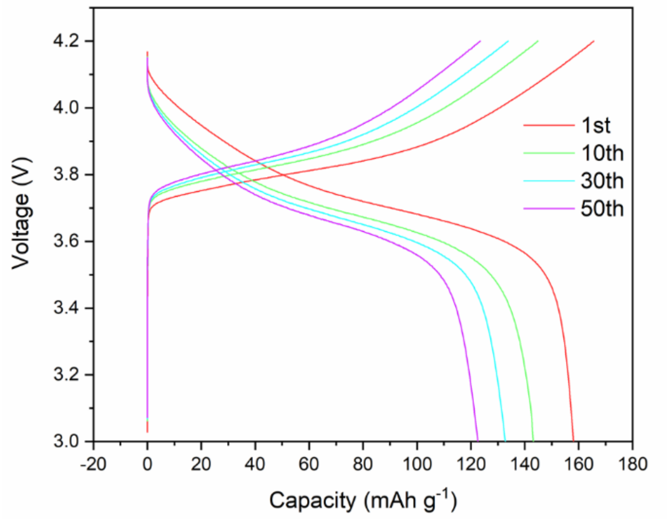


**Fig. S25** Corresponding voltage profile of Li/CMP/VAMMT/NCM523 cell at the 1, 10, 20, 40 cycles

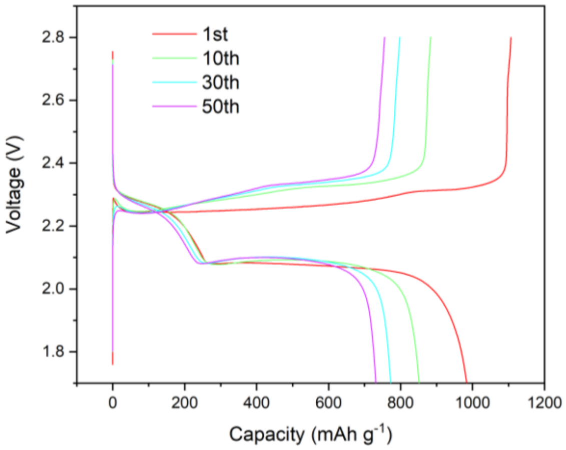


**Fig. S26** Corresponding voltage profile of Li/CMP/VAMMT/S cell at the 1, 10, 30, 50 cycles

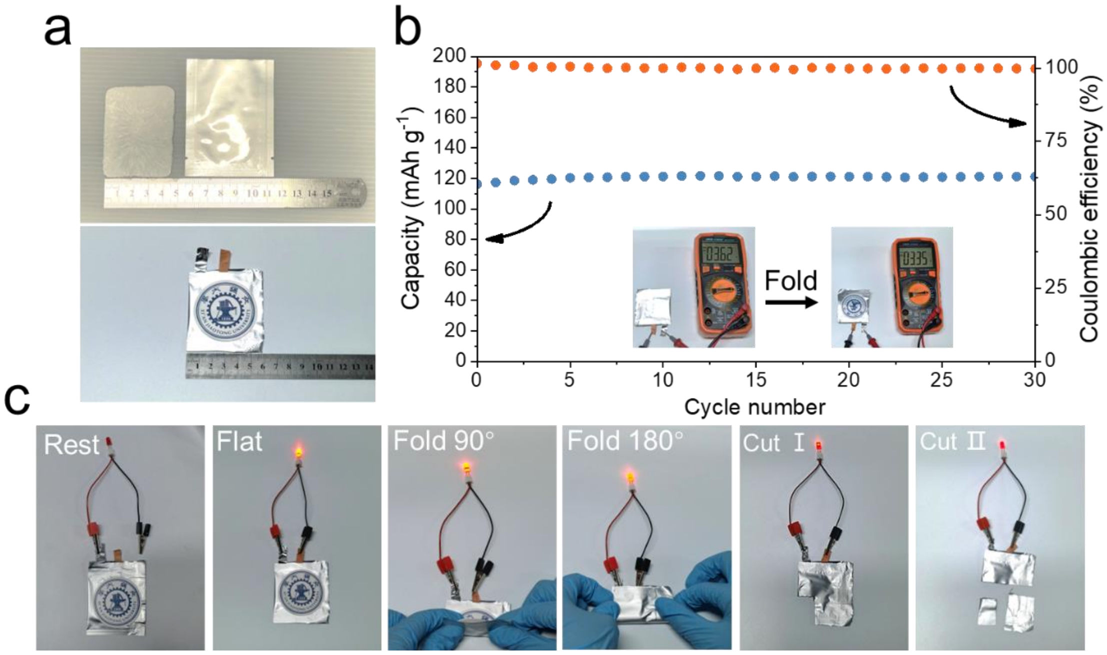


**Fig. S27** Digital photo of larger size VAMMT (above) and Li//LFP pouch cell with CMP/VAMMT (bottom) (**a**). Cycle performance of LFP/CMP/VAMMT/Li pouch cell at 0.2 C, 30°C (**b**). optical images of folding and cutting test of pouch-type LFP/CMP/VAMMT/Li cell (**c**)

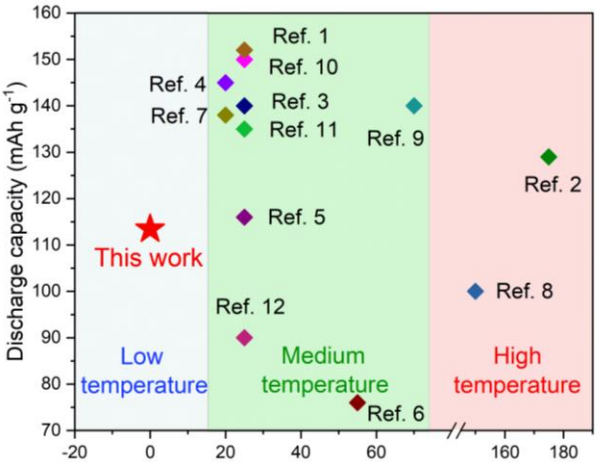


**Fig. S28** Capacity performance of the CMP/VAMMT operating at low temperature compared with other gel electrolytes reported in the literature

**Table S1** Comparison of the comprehensive performance of pure CMP, CMP/VAMMT and solid polymer electrolyte (SPE) in the literature

| | **Pure CMP** | **CMP/VAMMT** | **SPE [S1]** |
|---|---|---|---|
| Conductivity (RT) | Good | Very good | Medium |
| Mechanical strength | Medium | Good | Very good |
| Safety | Medium | Good | Very good |
| Operating temperature (lower limit) | Good | Very good | Medium |
| Stability against Li metal | Medium | Very good | Very good |
| Transference number | Good | Very good | Medium |

**Table S2** Comparison of cycling performance of full cell with different gel electrolyte at different temperatures in the Fig. S28

| **Electrolyte** | **Cycling temperature (°C)** | **Cathode** | **Test rate (C)** | **Average discharge capacity (mAh g$^{-1}$)** | **Refs.** |
|---|---|---|---|---|---|
| **This work** | **0** | $LiFePO_4$ | **0.2** | **~115** | |
| P(VDF-HFP)+ZnS-NHIF/1.0 M LiTFSI/EC+DMC (1:1, vol%) | 25 | $LiFePO_4$ | 0.1 | ~150 | [S2] |
| P(VDF-HFP)+P(ETPTA)/3 M LiFSI/SN | 25 | $LiFePO_4$ | 1 | ~129 | [S3] |
| hBN/1.0 M LiTFSI/EMIM-TFSI | 175 | $LiFePO_4$ | 10 | ~140 | [S4] |
| BC+LLTO NWs/1.0 M $LiPF_6$/ EC+DMC (1:1, vol%) | RT | $LiFePO_4$ | 0.2 | ~145 | [S5] |
| P(EPTPA-co-PEGDA)/LiTFSI+$LiPF_6$ (1:1, molar ratio)/ EC+DMC+DEC (1:1:1, vol%) | 20 | $LiFePO_4$ | 0.5 | ~116 | [S6] |
| P(MPC-co-SBVI)/1.0 M LiTFSI/BMP-TFSI | RT | NCM523 | 1 | ~76 | [S7] |
| PDMA-silica/2.75 M LiTFSI/G4 | 55 | $LiFePO_4$ | 1 | ~138 | [S8] |
| P(VDF-HFP)+LLZO-Ga/LiFSI/TEP+FEC (7:3, vol%) | 20 | NCM523 | 0.5 | ~100 | [S9] |
| 10 wt% PBDT/10 wt% LiTFSI/80 wt% $Pyr_{14}$TFSI | 150 | $LiFePO_4$ | 1 | ~140 | [S10] |
| PVDF/LiBOB+LiTFSI (3:2, molar ratio)/EC+FEC+PC (49:49:2, vol%) | 70 | $LiFePO_4$ | 1 | ~152 | [S11] |
| P(VDF-HFP)+P(ETPTA)+MMT/1.0 M $LiPF_6$/EC+DEC (1:1, vol%) | 25 | $LiCoO_2$ | 0.5 | ~135 | [S12] |
| P(DOL)/2.0 M LiTFSI | RT | $LiFePO_4$ | 1 | ~90 | [S13] |

## Supplementary References

[S1] Z.J. Sun, Y.H. Li, S.Y. Zhang, L. Shi, H. Wu et al., G-$C_3N_4$ nanosheets enhanced solid polymer electrolytes with excellent electrochemical performance, mechanical properties, and thermal stability. J. Mater. Chem. A **7**(18), 11069-11076 (2019). https://doi.org/10.1039/c9ta00634f

[S2] P. Bose, D. Deb, S. Bhattacharya, Lithium-polymer battery with ionic liquid tethered nanoparticles incorporated P(VDF-HFP) nanocomposite gel polymer electrolyte.

Electrochim. Acta **319**, 753-765 (2019). https://doi.org/10.1016/j.electacta.2019.07.013
[S3] W. Zha, J. Li, W. Li, C. Sun, Z. Wen, Anchoring succinonitrile by solvent-$Li^+$ associations for high-performance solid-state lithium battery. Chem. Eng. J. **406**, 126754 (2021). https://doi.org/10.1016/j.cej.2020.126754
[S4] W.J. Hyun, A.C.M. Moraes, J.M. Lim, J.R. Downing, K.Y. Park et al., High-modulus hexagonal boron nitride nanoplatelet gel electrolytes for solid-state rechargeable lithium-ion batteries. ACS Nano **13**(8), 9664-9672 (2019). https://doi.org/10.1021/acsnano.9b04989
[S5] C. Ding, X. Fu, H. Li, J. Yang, J.L. Lan et al., An ultrarobust composite gel electrolyte stabilizing ion deposition for long-life lithium metal batteries. Adv. Funct. Mater. **29**(43), 1904547 (2019). https://doi.org/10.1002/adfm.201904547
[S6] W. Fan, N.W. Li, X. Zhang, S. Zhao, R. Cao et al., A dual-salt gel polymer electrolyte with 3D cross-linked polymer network for dendrite-free lithium metal batteries. Adv. Sci. **5**(9), 1800559 (2018). https://doi.org/10.1002/advs.201800559
[S7] A.J. D'Angelo, M.J. Panzer, Decoupling the ionic conductivity and elastic modulus of gel electrolytes: fully zwitterionic copolymer scaffolds in lithium salt/ionic liquid solutions. Adv. Energy Mater. **8**(26), 1801646 (2018). https://doi.org/10.1002/aenm.201801646
[S8] L. Yu, S. Guo, Y. Lu, Y. Li, X. Lan et al., Highly tough, Li-metal compatible organic–inorganic double-network solvate ionogel. Adv. Energy Mater. **9**(22), 1900257 (2019). https://doi.org/10.1002/aenm.201900257
[S9] D. Xu, J.M. Su, J. Jin, C. Sun, Y.D. Ruan et al., In situ generated fireproof gel polymer electrolyte with $Li_{6.4}Ga_{0.2}La_3Zr_2O_{12}$ as initiator and ion-conductive filler. Adv. Energy Mater. **9**(25), 1900611-1190622 (2019). https://doi.org/10.1002/aenm.201900611
[S10] D.Y. Yu, X.N. Pan, J.E. Bostwick, C.J. Zanelotti, L.Q. Mu et al., Room temperature to 150 °C lithium metal batteries enabled by a rigid molecular ionic composite electrolyte. Adv. Energy Mater. **11**(12), 2003559 (2021). https://doi.org/10.1002/aenm.202003559
[S11] J. Yu, J. Liu, X. Lin, H.M. Law, G. Zhou et al., A solid-like dual-salt polymer electrolyte for Li-metal batteries capable of stable operation over an extended temperature range. Energy Storage Mater. **37**, 609-618 (2021). https://doi.org/10.1016/j.ensm.2021.02.045
[S12] Y.M. Jeon, S. Kim, M. Lee, W.B. Lee, J.H. Park, Polymer-clay nanocomposite solid-state electrolyte with selective cation transport boosting and retarded lithium dendrite formation. Adv. Energy Mater. **10**(47), 2003114 (2020). https://doi.org/10.1002/aenm.202003114
[S13] Q. Zhao, X. Liu, S. Stalin, K. Khan, L.A. Archer, Solid-state polymer electrolytes with in-built fast interfacial transport for secondary lithium batteries. Nat. Energy **4**(5), 365-373 (2019). https://doi.org/10.1038/s41560-019-0349-7

## Author contributions

X.L., Y.W., K.X. performed the experiments and co-wrote the paper. X.L., Y.W., K.X. and S.D. conceived the idea, planned the study, designed the experiment, analysed the data and composed the manuscript. X.L. performed all of the experiments with the assistance of J.F., G.Z., W.H. and W.Y., H.W. directed the revision of the article. Q.J. and A.A. supervised the project. All of the authors reviewed and commented on the manuscript.
